# Examination of the structural components of the Abilitator–A self-report questionnaire on work ability and functioning aimed at the population in a weak labour market position

**DOI:** 10.1371/journal.pone.0300182

**Published:** 2024-03-19

**Authors:** Miia Wikström, Anne Kouvonen, Jouko Remes, Kaisa Törnroos, Matti Joensuu

**Affiliations:** 1 Work Ability and Working Life, Finnish Institute of Occupational Health, Helsinki, Finland; 2 Faculty of Social Sciences, University of Helsinki, Helsinki, Finland; 3 Centre for Public Health, Queen’s University Belfast, Belfast, United Kingdom; 4 ICT and Digital Services, Finnish Institute of Occupational Health, Oulu, Finland; University of Maribor, SLOVENIA

## Abstract

**Objectives:**

According to the Consensus-based Standards for the selection of health Measurement Instruments (COSMIN) panel, structural validity describes how well Patient-Reported Outcome Measures’ (PROM) scores reflect the dimensions of the measured construct. The main purpose of this study was to examine the structural components of the Abilitator, a co-developed self-report questionnaire on work ability and functioning for the population in a weak labour market position.

**Methods:**

We examined to what extent the Abilitator has reflective and formative elements in its five summary scales: “C. Inclusion”, “D. Mind”, “E. Everyday life”, “F. Skills”, and “G. Body”. The Abilitator data sample (n = 4555, men 51%, mean age 37 years) was collected in 2017–2022 by the Finnish Institute of Occupational Health in cooperation with the European Social Fund Priority 5 projects in which the participants have multiple challenges to gain employment. For the structural components and validity analysis we implemented both Confirmatory Factor Analysis (CFA) and Exploratory Factor Analysis (EFA).

**Results:**

Based on the COSMIN criteria for structural validity, the Abilitator reached approximate model fit with CFA when we analysed the different concepts of the questionnaire separately rather than in one unified model. An exception was “E. Everyday life” which was a formative summary scale, and it did not reach approximate fit. EFA showed that the items in the Abilitator’s summary scales loaded on ten factors.

**Conclusions:**

The Abilitator had both reflective and formative elements in its structure. It reached structural validity in those separate concepts that were based on a reflective model. This study revealed interesting connections between different aspects of the Abilitator and produced valuable information for further modification of the questionnaire.

## Introduction

The population’s health and quality of life would both improve if there were less unemployment [1]. Minimizing long-term unemployment could also increase social inclusion and equality in working life and reduce marginalization [2–4]. Therefore, measures to promote employment should be a societal priority [1].

However, many working-age people face persistent difficulties finding sustainable employment. This may be due to a migrant background, low-level education or skills, short work history, chronic health problems, disabilities, prolonged unemployment, living in a rural area, or being young or old [5–7]. These people are in a weak labour market position and could benefit from individually designed measures to support their participation in employment [6, 8].

In most Western European welfare states, the working-age population has access to a wide range of services to support their employment, education, health, and well-being. To assess which services might be the most suitable for each individual, we need validated, multi-professionally usable, quickly managed, and easily interpreted instruments. Alongside non-patient-reported assessments carried out by clinicians or service professionals, the use of Patient-Reported Outcome Measures (PROMs) has become popular [9–12]. PROMs are self-report questionnaires for individuals to assess their own situation in terms of health, health-related quality of life, and life situation [13–18]. The work ability and functioning of the unemployed are important aspects to consider as they are known to have poorer health and work ability than those who are in employment [19–21]. For example, in Finland, health and work ability problems are estimated to be an obstacle to employment for 45% of the unemployed population [5].

Work ability is a multidimensional concept that combines health, functioning, basic skills, and the occupationally relevant attributes required for executing work tasks in an acceptable work environment [22, 23]. It also contains elements related to the individuals’ social networks and opportunities in their living environment [23]. Functioning is integrally linked to health and includes psychological, social, physical, and cognitive dimensions [24, 25].

At present only a few validated PROMs for the multidimensional assessment of work ability and functioning are aimed at the population in a weak labour market position [26–28]. The Abilitator® self-report instrument was developed in part to fill this gap. The Abilitator is a digital questionnaire that produces an individual report with written feedback and suggests further actions for maintaining or improving work ability and functioning. It is an indicative rather than a diagnostic instrument. It can be described as a “resource-oriented work ability mapping tool” for the general, non-observable work ability-related aspects that should be considered when building one’s path towards employment [29]. On the one hand, the Abilitator helps unemployed individuals identify their strengths and challenges in terms of their work ability and functioning. On the other hand, by producing valuable information it helps service professionals suggest the most effective means to support their clients’ transition towards employment [29, 30].

A PROM can be considered of high-quality when it is valid, reliable, responsive, and interpretable [31]. Previous studies on the Abilitator’s content validity [29], concurrent validity [32], and intrarater test-retest reliability [33] have supported the view that the Abilitator is a high-quality self-report questionnaire. However, further evidence of its psychometric properties is required. By examining the internal structure of the Abilitator we can obtain further information on how the different concepts and items in the instrument are related to each other.

The Abilitator’s conceptual model on work ability, i.e., the theoretical model of how different constructs within its concepts are related [31] is based on the multi-dimensional theory of the Work Ability House [23]. This theory constructs work ability as the dynamic balance between individual-related resources such as health, functioning, motivation and skills, and the operational environment such as social networks and service structure [23, 34]. The model also covers elements related to work, working conditions, colleagues, and leadership. However, the Abilitator does not include these work-related aspects, because the majority of individuals in a weak labour market position are not in employment. Within work ability, the conceptual model of functioning is viewed as biopsychosocial [24].

In addition to being a self-report tool for work ability and functioning, the Abilitator was developed for a variety of other purposes, including setting individual goals in different areas of life, and tracking their achievement. To meet these needs, the Abilitator combines both existing questionnaires and some new questions into one self-report questionnaire [29]. The Abilitator’s conceptual framework i.e., the model representing the relationships between the items and the construct to be measured [31] has both reflective and formative elements. This means that the underlying model for some of the constructs in the Abilitator are reflected by all its items, and other constructs are formed because of the items they include. The items in the formative constructs do not always correlate with each other [31, 35]. For example, many different situations and circumstances might affect concepts such as coping in everyday life, but they do not all need to exist at the same time.

A measurement theory describes how the scores produced by the items represent the construct to be measured [31]. This theory is especially important with multi-item instruments, which contain several indirectly measured unobservable items [31]. The Abilitator’s underlying measurement theory is the Classical Test Theory (CTT) [36]. This means that in the constructs that are based on a reflective model, the information is obtained by measuring the items that display the construct. However, no well-developed theories are available for the constructs that are based on a formative model. These constructs are based on common sense [31].

According to the Consensus-based Standards for the selection of health Measurement Instruments (COSMIN) panel’s taxonomy, construct validity is one of the main elements when assessing a PROM’s measurement properties [37]. Construct validity is defined as the degree to which the scores of a measurement tool are consistent with the presumed construct in terms of the internal relationships, with the scores of other tools, or with the differences between the groups [31, 37]. It is assessed when no gold standard is available [31] and when abstract variables such as social inclusion or coping with everyday life are observed [38].

Structural validity is a subtype of construct validity [31]. It describes how well the PROM’s scores reflect the dimensions of the measured construct [37]. It has been proposed that structural validity is not pertinent for all types of PROMs [35]. When a self-report tool is based on a reflective model and has effect indicators, i.e., items that are highly correlated, interchangeable and all indicate the same underlying construct, the assessment of structural validity is important. In contrast, structural validity is not relevant when a PROM is based on a formative model in which the items form the construct and are not necessarily correlated [35].

As the Abilitator has both formative and reflective elements in its conceptual framework, a structural validity assessment is not essential. However, we wanted to determine how reflective the Abilitator’s summary scale elements are. It has also been recommended that when an instrument already exists and has a mixed conceptual framework, it should be considered a reflective model [35].

The overall aim of this study was to examine the internal structure and structural validity of the Abilitator. This aim was achieved.

## Materials and methods

### The Abilitator self-report questionnaire

The Abilitator was co-developed in 2014–2017 at the Finnish Institute of Occupational Health (FIOH) in “The Social Inclusion and the Change of One’s Work Ability and Capacity” (Solmu), a national coordination project funded by the European Social Fund (ESF) Priority 5 programme (2014–2023) [29, 32, 33, 39]. One of the main aims of the ESF Priority 5 programme was to improve the social inclusion, work ability, and functioning of those in a weak labour market position [40]. Solmu’s target was to jointly develop a PROM ‘The Abilitator’, which would evaluate both the work ability and functioning of those participating in the national Priority 5 projects and detect any changes after the projects [29]. In addition to the ESF projects in Finland, the Abilitator has been used in research [8, 41, 42] and in employment, health, and social services to support primary-level decision-making when large numbers of clients meet professionals with various occupational backgrounds [43, 44].

**Fig 1:**
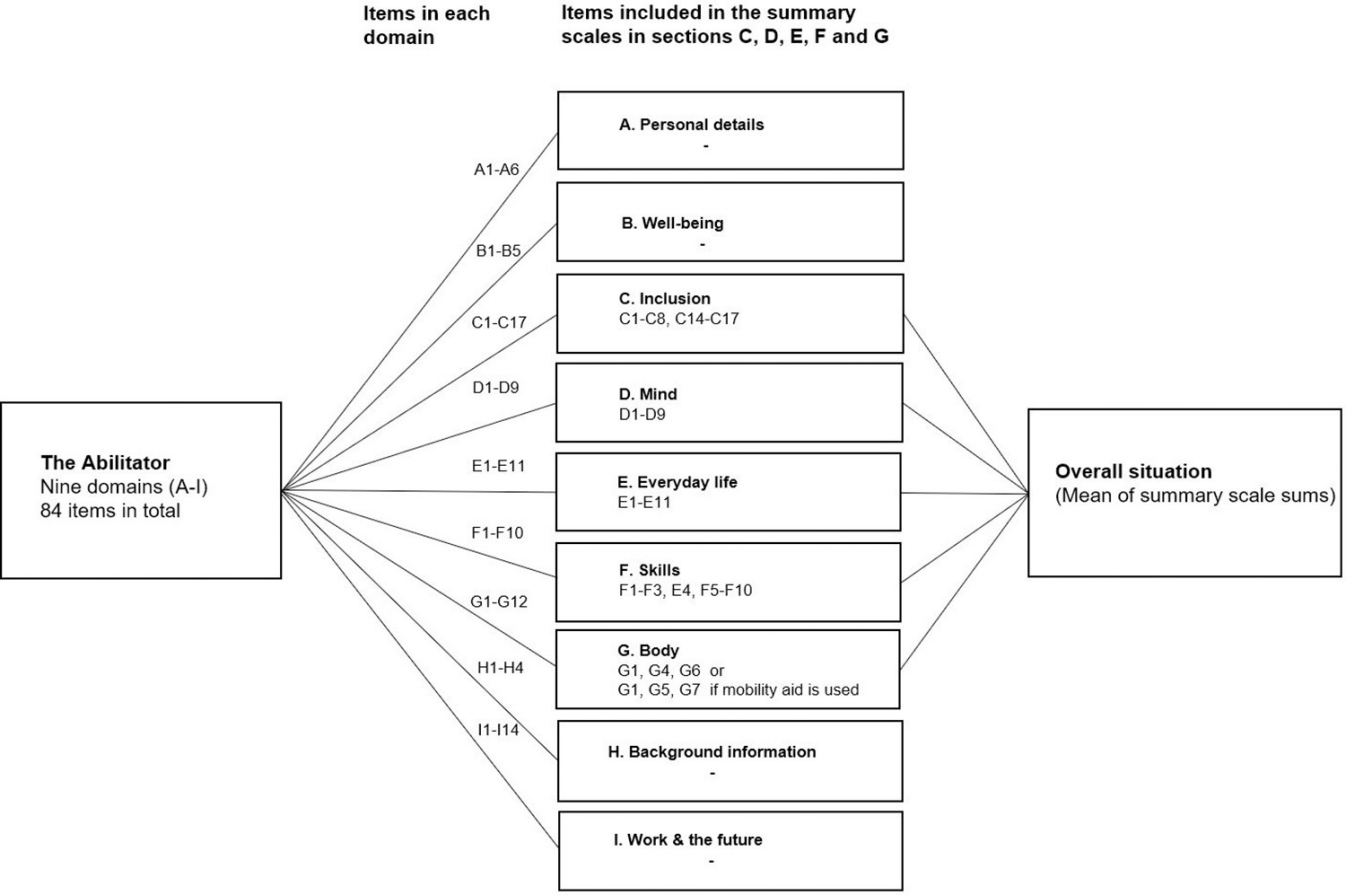
The Abilitator’s domains, total number of items in each domain, items included in the summary scales, and summary scales included in the “Overall situation” score [33].

The Abilitator contains 84 items under nine domains (sections): “A. Personal details”, “B. Well-being”, “C. Inclusion”, “D. Mind”, “E. Everyday life”, “F. Skills”, “G. Body”, “H. Background information”, and “I. Work and the Future” [29, 32, 33]. Each section contains 4–14 items (S1 Appendix). The measure of each of the five reported sections C, D, E, F, and G is a summary scale, transformed into a score of 0% to 100% of the selected items (Fig 1, Table 1). The smallest detectable changes in the Abilitator’s summary scale scores are presented in Wikström et al. [33]. The qualitative data is gathered from all the questions in sections A, H, I, and in separate questions of C9-C13, F4, G2, G3, G8, G9-G12. These questions are valuable for the respondents to reflect on, but they also provide useful additional information on the respondent’s situation for the service professionals.

**Table 1:** Interpretation of the Abilitator’s results based on the summary scales included in the “Overall situation” score [30].

	Poor (%)	Fairly poor (%)	Fairly good (%)	Good (%)
**C. Inclusion**	0–23	25–48	50–73	75–100
**D. Mind**	0–22	25–56	58–70	71–100
**E. Everyday life**	0–23	25–48	50–73	75–100
**F. Skills**	0–48	50–60	61–73	75–100
**G. Body**	0–30	40–60	61–80	90–100

These sections are numerically valued on a scale of 0–100%

In this study, we examined the structural components of the summary scales formed from sections C, D, E, F, and G, which contain the dimensions of social functioning and participation, psychological functioning, managing in everyday life, cognitive functioning and basic skills, and physical functioning, respectively. The “Overall situation” score, which is the mean of the sums obtained from these five summary scales, was not included in the analysis. There are no limit values for this score and the obtained data is used by service professionals to monitor the overall change in the respondent’s work ability and functioning, for example, when the Abilitator is applied for the second time after an intervention. Also, sections A, H, and I were excluded from the analysis since they were developed to provide background information and do not form a summary scale. Even though the Abilitator is available in 10 different languages, in this study we only examined the original Finnish version.

### Study sample

The entire Abilitator data (n = 54464) has been collected by FIOH in various services and projects aimed mainly for the unemployed. To ensure that the participants in this study were in a weak labour market position, the sample utilized in this study (n = 4555) was collected in cooperation with the European Social Fund Priority 5 projects in 01.10.2017–31.12.2022. Each project (n = 150) gave written consent to FIOH to use their Abilitator data in an anonymous format in its research. All the Abilitator respondents in the data were participants in the ESF Priority 5 projects, which aim to improve the work ability and functioning of people not in employment to help them proceed on employment paths and to strengthen social inclusion [40]. In each project the participants took part in activities such as cooking, gardening, social interaction, light physical activity, and rehabilitative work tasks. The participants could choose whether to answer the questionnaire online via the Abilitator online service or in a paper format. In the latter case, the staff of each ESF project transferred the answers to the Abilitator online service with the permission of the respondent.

### Research design

The structural examination of the Abilitator followed the guidelines of the COSMIN panel [31, 35]. These guidelines recommend the implementation of factor analysis (FA) to determine the dimensionality of the data based on item correlations [31]. In FA, the items that are highly correlated with each other can be grouped in one factor. The purpose of FA is to find the number of meaningful factors in data or in a construct. Of the FA types, the use of exploratory factor analysis (EFA) is suggested if the number of the dimensions in the instrument is not known beforehand. The use of confirmatory factor analysis (CFA) is recommended if the dimensions of the instrument are predetermined [31]. Because the latter was the case with the Abilitator, we carried out CFA to determine whether the collected data fits the assumed factor structure. In addition to this strictly confirmatory approach, an alternative model approach was taken to examine other models that would fit the Abilitator’s factor structure well [45]. After CFA it was also relevant to conduct EFA to discover how the Abilitator’s items loaded freely. We used Mplus software version 8 to conduct the CFA and EFA [46]. The same data sample was used for all the analyses, because EFA was not used to develop a reflective, unidimensional, data driven model, which should be confirmed in a separate sample.

### Data analysis

In the CFA, we followed three phases: 1) preparation, 2) model testing and 3) reporting the results [47]. In the first phase, we prepared the data for the actual CFA analysis. The Abilitator data we used (n = 4555) consisted solely of the data collected from the ESF Priority 5 projects that had two or more participants. This included the individuals who had responded to the Abilitator in Finnish and were between 18 and 74 years old i.e., belonged to the labour force in Finland. The respondents who regularly used an aid when moving around were excluded. We described the study population by age, gender, self-rated health, perceived general functioning, perceived work ability and duration of unemployment.

After these preparations, we screened the data by clarifying the sample size and its variation. The recommended sample size for CFA is 3 to 15 times the number of variables [48]. To ensure sufficient variation, the data set should be over 400 cases, especially when the maximum likelihood with robust standard errors (MLR) method is used, with continuous non-normal data containing missing values [49]. We also searched the data for and reported missing response percentages and checked for multivariate outliers. In addition, we analysed the data set for possible floor and ceiling effects. These effects may occur if more than 15% of all responses score at the lower or upper end of the scales [31]. Both CFA and later EFA were conducted with multilevel modeling which considers the clustering of participants in specific ESF projects (TYPE = COMPLEX). Missing values were handled with the Missing data at random (MAR) option in Mplus. [46]. For EFA the type of rotation used was GEOMIN i.e., oblique rotation where the dimensions of the Abilitator were allowed to correlate [45, 46].

In the second phase of CFA, we conducted the model testing, which was further divided into five sub-phases: 1) specification, 2) identification, 3) estimation, 4) evaluation, and 5) modification [47]. In the specification phase, we selected the first model to be tested on the basis of the hypothesis that the summary scales of the Abilitator comprise 5 concepts and 44 selected items. We further selected the second set of models on the basis of the assumption that the Abilitator consists of five separate concepts with 3–12 items within each concept.

In the identification phase, we identified the tested models by fixing one loading in each concept (factor) to one as a reference variable and the rest to zero. Only those models that were identified could be estimated. If the model’s degrees of freedom (df) were over 0 the model was identified and if 0 it was just identified. If the model’s df was under 0 it was not identified.

In the estimation phase, we estimated the factor loadings and residual error terms for all the models using the MLR method (ESTIMATOR = MLR). In the evaluation phase, we analysed how well the selected models matched the data using five indicators: the Comparative Fit Index (CFI), the Tucker-Lewis Index (TLI), the Root Mean Square Error of approximation (RMSEA), the Standardized Root Mean Square Residual (SRMR) and the Chi-square (χ^2^) test. Of these, CFI and TLI are incremental fit indices and utilize the worst possible model (null model) as a basis for assessing how well the model fits [45]. The other extreme is the best possible model (saturated model). RMSEA, SRMR and χ^2^ are absolute fit indices for the whole model and are not compared with the null model [45]. The COSMIN cut-off limits for good model fit and measurement properties in CFA are CFI ≥0.95, TLI ≥0.95, RMSEA ≤0.06, SRMR ≤0.08, and χ^2^ should be p ≥0.05 [35]. However, if the measurement instrument is based on CTT, only one of these listed limits must be met to reach good measurement properties. In addition, the result of the χ^2^ test is not required [35].

In the modification phase, we further improved the tested models by utilizing the result of the EFA analysis, in which the data was allowed to load freely, and with the modification index (MI). MI is related to assessing the fixed parameters and is a tool for determining potential sources of model misfit [45]. With the knowledge of the highest MIs between the variables, we allowed some of the error terms to correlate to improve the model fit. For the EFA, factor loadings of ≥0.30 were considered substantial [31].

In the third phase of the CFA analysis, we reported the results for all the tested models with standardized values for factor loadings and error terms, correlations between concepts, and MIs in both graphic form and tables. The strength of the standardized factor loadings was considered sufficiently high if ≥0.5, but ideal if ≥0.7 [50]. For CFA we used STYDYX standardization which is recommended for continuous variables [46].

### Ethics statement

The study was approved by the ethics board of the Finnish Institute of Occupational Health in June 2017. All the respondents had been given written information about the Abilitator’s data security and the possible later research use of the data in anonymous format. They had also all consented to this by voluntarily responding to the Abilitator self-report questionnaire.

## Results and discussion

### Study population characteristics

The study population (N = 4555, 51% men, 47% women and 2% other, mean age 37 years) was made up of participants of 150 different ESF Priority 5 projects (Table 2). Most had problems with their health, general functioning, and work ability. Eighteen per cent of the participants had been unemployed for less than a year, 21% for one to two years, 34% for three to ten years and 9% for over 10 years. Nine per cent of the participants had never worked and another 9% were in employment at the time of the questionnaire. In terms of the Abilitator’s score categories (Table 1), 23% of the participants had a poor or fairly poor situation in section “C. Inclusion”, 42% in “D. Mind”, 7% in “E. Everyday life”, 37% in "F. Skills”, and 37% in "G. Body”, respectively.

**Table 2:** Study population characteristics.

	ESF Priority 5 (n = 4555)
	n (%)
**Gender**	Men 2319 (51)
	Women 2159 (47)
	Other 77 (2)
**Age in years mean (min–max)**	37.2 (18–72)
**B2. Self-rated health**	3.17
**Mean (95% CI of mean, SD)**	(3.14–3.20, 1.04)
Poor or fairly poor (1–2 points)	1187 (26.1)
Average (3 points)	1691 (37.1)
Fairly good (4 points)	1157 (25.5)
Good (5 points)	516 (11.3)
**B3. Perceived general functioning**	6.54
**Mean (95% CI of mean, SD)**	(6.47–6.60, 2.15)
Poor (0–5 points)	1412 (31)
Fairly poor (6–7 points)	1398 (30.7)
Good (8–9 points)	1454 (31.9)
Excellent (10 points)	291 (6.4)
**B4. Perceived work ability**	5.75
**Mean (95% CI of mean, SD)**	(5.68–5.82, 2.53)
Poor (0–5 points)	1975 (43.4)
Fairly poor (6–7 points)	1295 (28.4)
Good (8–9 points)	1072 (23.5)
Excellent (10 points)	213 (4.7)

ESF, European Social Fund; CI, Confidence Interval; SD, Standard Deviation.

### Confirmatory and exploratory factor analysis

In terms of variation, the sample size was large enough for CFA to be conducted. The requirement was either over 400 or 3–15 times the number of the analysed 44 variables i.e., 132 to 660. The percentages of missing values were 0.92% for the whole sample, 0.76% for “C. Inclusion, 1.81% for “D. Mind”, 0.73% for “E. Everyday life”, 0.52% for “F. Skills”, and 0.45% for “G. Body”. No floor or ceiling effects were found, as less than 15% of the responses per analysed item or summary scale scored at the lowest or highest score of the scale. Therefore, the data was considered continuous and normally distributed. In addition, there were no outliers in the sample since all items could be scored from 1 to 5.

**Fig 2:**
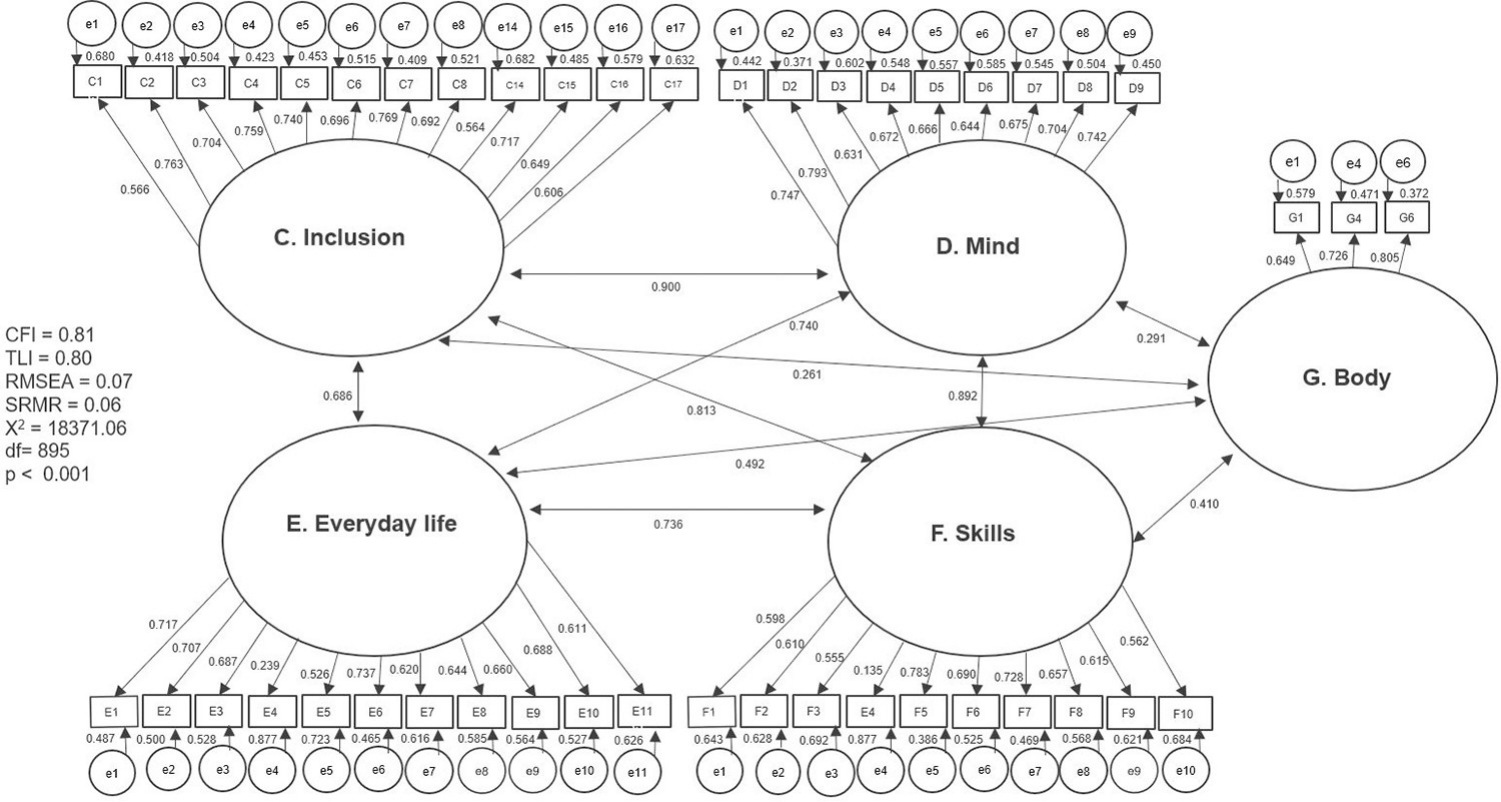
Model with all the Abilitator’s five concepts together.

**Fig 3:**
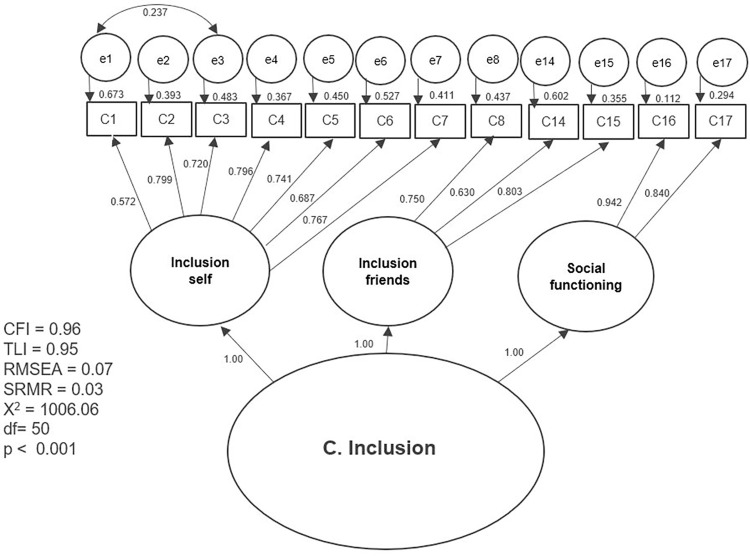
Separate model with the Abilitator’s concept “C. Inclusion” with partial modifications.

**Fig 4:**
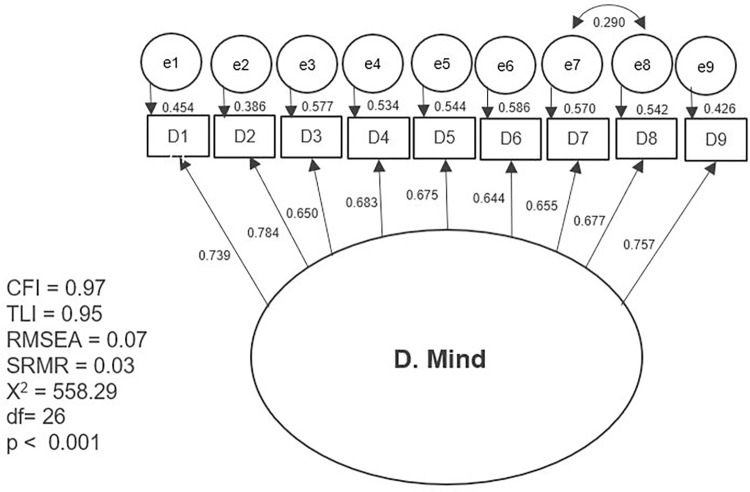
Separate model with the Abilitator’s concept “D. Mind”.

**Fig 5:**
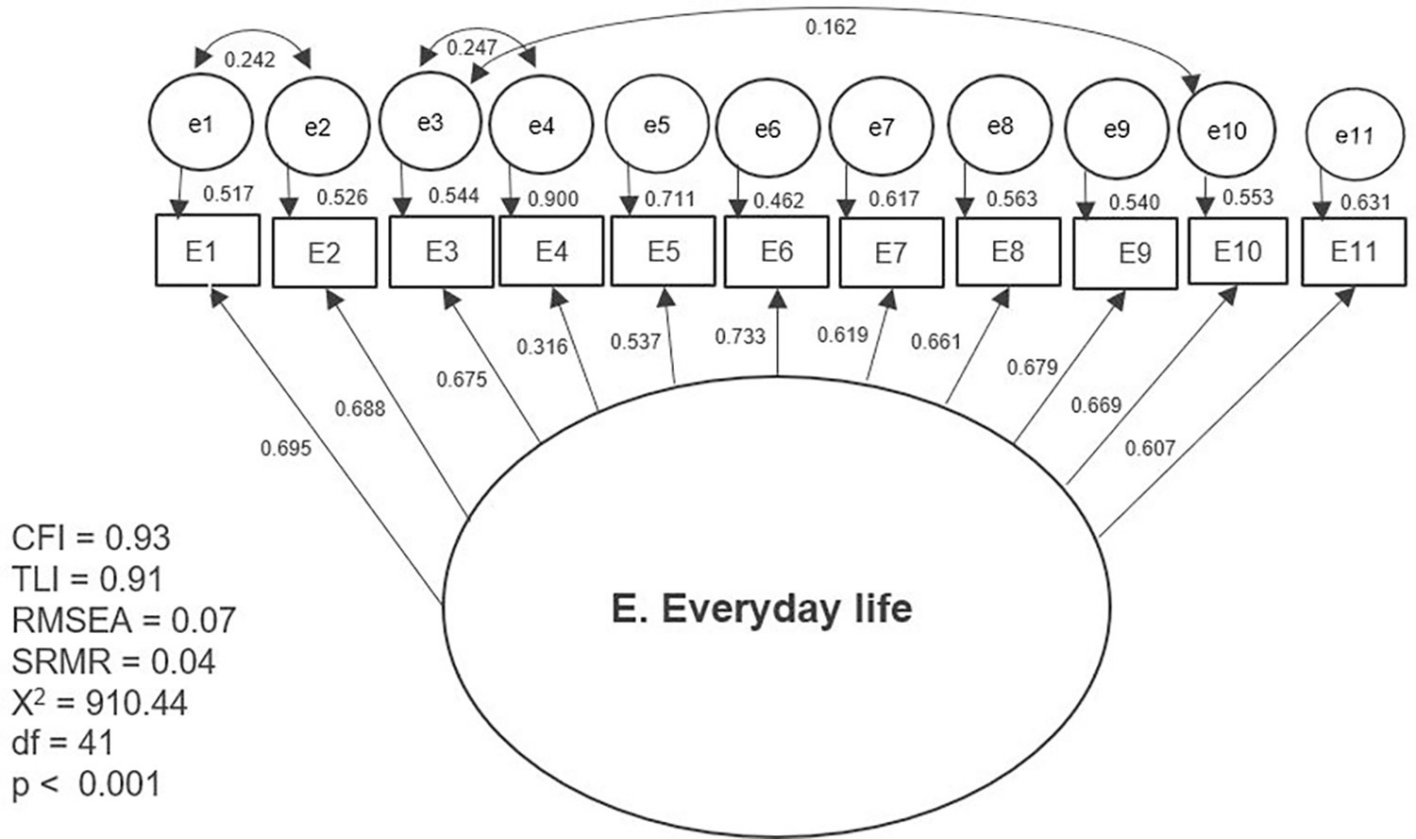
Separate model with the Abilitator’s concept “E. Everyday life”.

**Fig 6:**
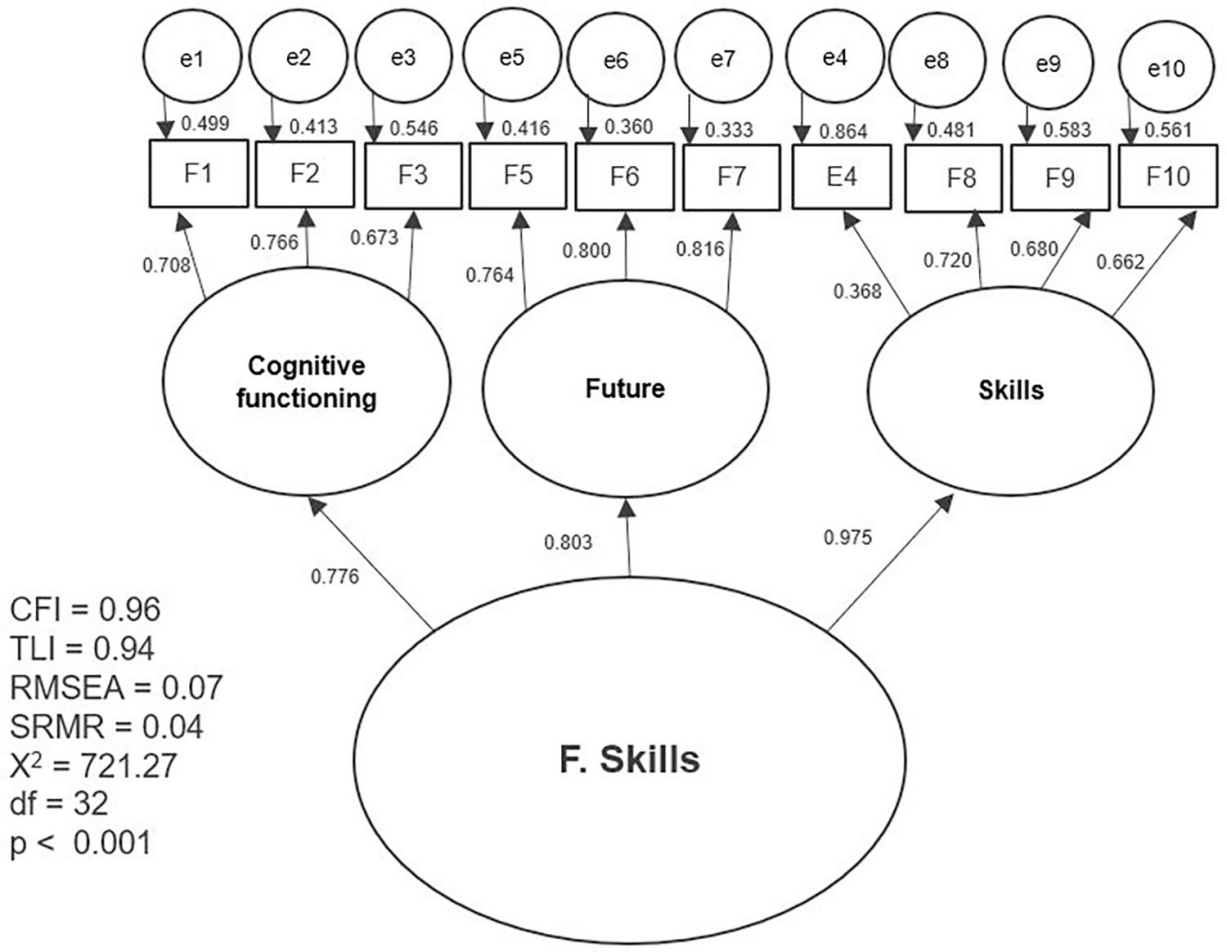
Separate model with the Abilitator’s concept “F. Skills” with partial modifications.

**Fig 7:**
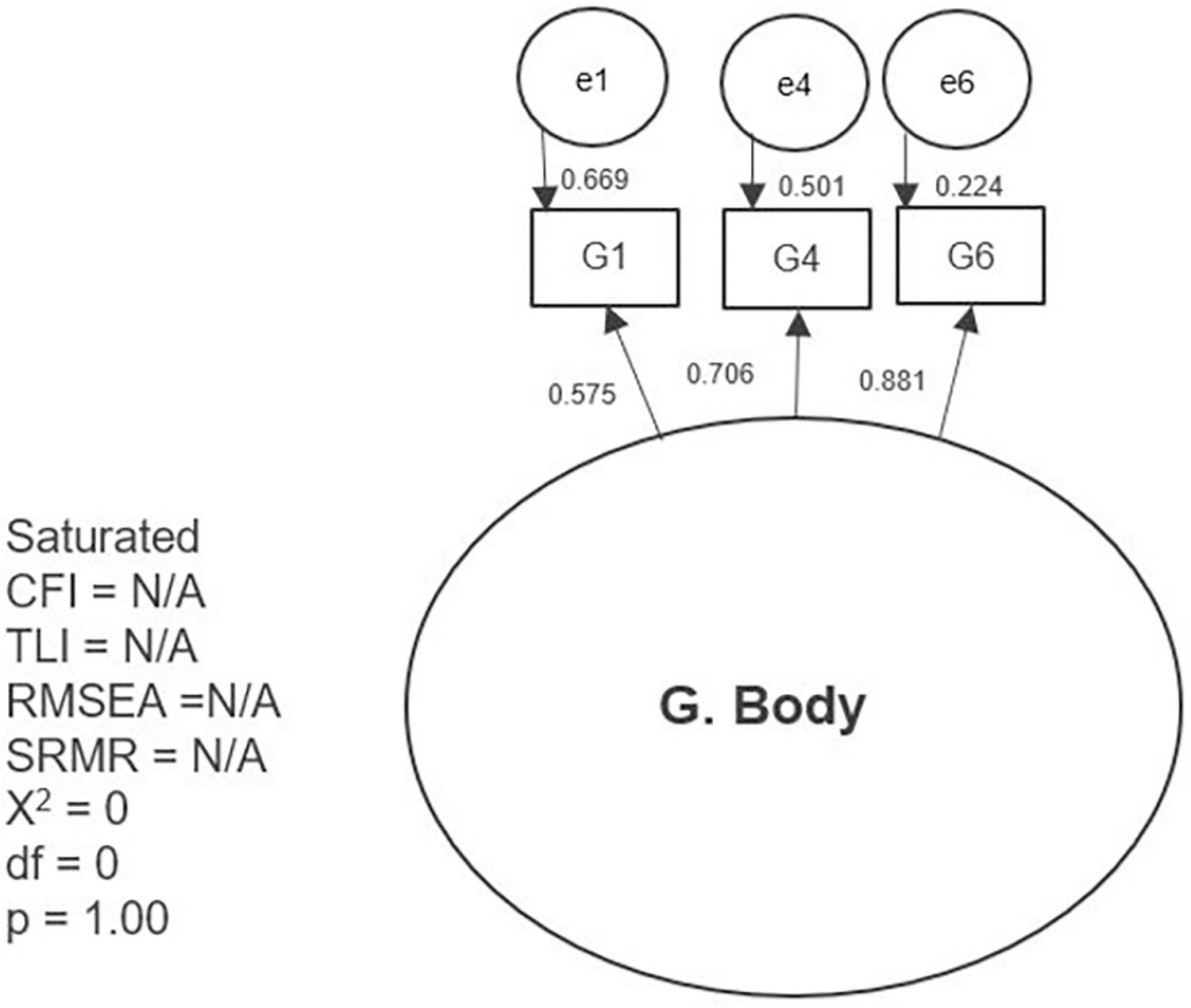
Separate model with the Abilitator’s concept “G. Body”.

The results of the first tested model and the separate models with partial modifications are presented separately in graphic form (Figs 2–7). The ovals represent independent latent variables (concepts), and the circles are independent latent error terms. The rectangles are the dependent measured items. The two-way arrows represent the correlation or covariance between variables and one-way arrows the standardized, unidirectional factor loadings. The goodness of fit indices are presented beside each graph and in Table 3. The Table 3 also presents the goodness of fit indices for an alternative model of the “F. Skills” without the Abilitator’s item E4 and the EFA with ten factors, which was the first well-fitting model.

**Table 3:** Goodness of fit indices in models tested with Confirmatory Factor Analysis (CFA) and Exploratory Factor Analysis (EFA).

Model	χ^2^, p*	df	CFI	TLI	RMSEA	SRMR
**Five concepts in one model**	18314.19, <0.001	895	0.81	0.80	0.07	0.06
**C. Inclusion**	1006.06, <0.001	50	0.96	0.95	0.07	0.03
**D. Mind**	558.29, <0.001	26	0.97	0.95	0.07	0.03
**E. Everyday life**	910.44, <0.001	41	0.93	0.91	0.07	0.04
**F. Skills**	721.27, <0.001	32	0.96	0.94	0.07	0.04
**F. Skills without question E4**	447.47, <0.001	24	0.97	0.96	0.06	0.03
**G. Body**	0, <0.001, saturated	0	N/A	N/A	N/A	N/A
**EFA with ten factors**	3867.93, <0.001	551	0.96	0.94	0.04	0.01

χ^2^, Chi-squared test

p*, the pursued p-value for χ^2^ test >0.05; df, degrees of freedom; CFI, Comparative Fit Index; TLI = Tucker-Lewis Index; RMSEA, Root Mean Square Error of Approximation; SRMR, Standardized root mean Square Residual.

The Abilitator’s factor loadings with EFA are presented in Table 4. Most of the items in the original summary scales loaded substantially on one to four different factors, but items in “D. Mind” on six factors. Items D3, D9 and E4 did not load substantially on any of the 10 factors. Items C6, C15, D5, E3, F6, F7 and F9 loaded substantially on two factors.

**Table 4:** Exploratory factor analysis with ten factors.

	F1	F2	F3	F4	F5	F6	F7	F8	F9	F10
**C1**	**0.47**	-0.11	0.01	0.22	0.01	0.04	0.01	0.11	-0.02	0.14
**C2**	**0.74**	0.04	0.02	0.03	-0.10	0.01	-0.03	-0.05	0.10	-0.09
**C3**	**0.61**	0.01	-0.02	0.16	0.12	-0.10	-0.00	0.06	0.04	0.13
**C4**	**0.83**	-0.02	-0.03	0.11	-0.03	-0.08	0.01	0.01	0.04	0.01
**C5**	**0.57**	0.00	0.04	-0.04	0.10	0.17	0.01	0.00	0.03	-0.01
**C6**	**0.33**	-0.00	0.13	0.05	**0.32**	0.12	-0.03	0.06	-0.12	0.06
**C7**	**0.49**	0.11	0.19	0.05	0.05	0.11	0.00	-0.01	-0.11	-0.00
**C8**	0.27	0.09	-0.00	**0.49**	0.05	0.04	0.01	-0.01	-0.09	-0.02
**C14**	0.10	0.02	0.04	**0.58**	0.00	-0.01	0.07	-0.02	0.03	0.04
**C15**	0.04	**0.38**	0.11	**0.47**	0.02	-0.00	0.04	-0.00	-0.02	-0.07
**C16**	0.05	**0.87**	0.01	0.09	0.02	-0.00	-0.02	0.01	-0.01	-0.02
**C17**	-0.03	**0.84**	0.03	0.02	-0.03	0.00	0.01	-0.00	0.04	0.07
**D1**	0.03	0.04	-0.03	-0.06	0.05	**0.60**	0.07	0.02	-0.03	0.01
**D2**	**0.52**	0.09	0.06	-0.05	0.07	0.20	0.08	-0.04	-0.00	-0.12
**D3**	0.20	0.01	0.01	0.03	0.19	0.15	0.29	0.06	-0.22	-0.02
**D4**	0.08	0.06	0.04	0.03	**0.39**	0.12	0.11	-0.01	0.07	-0.06
**D5**	0.07	0.04	0.03	0.01	**0.44**	-0.01	**0.32**	-0.02	-0.01	0.01
**D6**	0.23	0.12	-0.08	0.30	0.11	0.14	0.02	-0.02	0.07	-0.04
**D7**	0.02	0.04	0.02	0.05	**0.71**	0.02	0.03	0.01	0.01	0.10
**D8**	-0.00	0.06	0.28	-0.02	**0.55**	0.05	-0.04	0.00	0.05	-0.05
**D9**	0.20	-0.02	0.01	0.12	0.28	0.24	0.12	0.00	-0.00	-0.02
**E1**	-0.06	-0.02	**0.63**	0.06	0.05	0.06	0.04	0.15	-0.07	-0.02
**E2**	-0.03	0.03	**0.71**	-0.01	-0.04	0.06	0.05	0.07	-0.09	0.24
**E3**	0.04	0.08	**0.67**	-0.08	0.06	-0.03	0.06	-0.06	0.03	**0.33**
**E4**	0.03	-0.16	0.20	0.01	0.02	-0.03	0.23	0.03	0.24	0.29
**E5**	0.03	-0.10	**0.44**	-0.01	0.25	-0.12	0.11	-0.05	0.01	-0.03
**E6**	0.05	-0.00	**0.53**	0.05	0.14	0.00	0.04	0.15	-0.02	-0.26
**E7**	0.09	-0.01	**0.47**	-0.02	-0.03	0.04	0.14	0.05	-0.01	-0.22
**E8**	-0.04	-0.05	**0.61**	0.11	0.08	-0.04	-0.05	0.01	0.09	-0.08
**E9**	0.09	0.03	**0.63**	-0.01	0.07	-0.02	-0.08	-0.07	0.09	0.01
**E10**	0.05	0.12	**0.61**	0.01	-0.09	0.07	-0.00	0.08	0.00	0.20
**E11**	0.01	0.02	**0.47**	0.24	-0.03	0.04	-0.02	-0.02	0.19	0.05
**F1**	-0.05	0.01	0.18	0.07	0.09	0.01	0.60	-0.07	-0.00	0.01
**F2**	-0.01	0.02	-0.03	0.03	-0.04	0.07	0.66	0.03	0.23	0.08
**F3**	0.02	-0.00	0.15	0.00	0.01	-0.04	0.60	0.03	0.01	-0.08
**F5**	0.27	-0.01	0.09	0.01	0.00	**0.61**	0.03	0.00	0.02	0.00
**F6**	0.02	-0.05	0.01	0.11	-0.03	**0.67**	-0.04	0.01	**0.37**	0.05
**F7**	-0.04	0.01	0.06	0.08	0.08	**0.53**	-0.01	0.01	**0.43**	-0.06
**F8**	0.13	0.15	-0.02	-0.04	-0.01	0.23	0.17	0.04	**0.38**	-0.04
**F9**	0.03	**0.46**	0.01	0.00	0.15	-0.01	0.08	0.02	**0.32**	0.02
**F10**	0.04	0.13	0.01	-0.05	0.12	0.04	0.22	0.06	**0.39**	0.06
**G1**	0.09	0.05	0.13	-0.00	0.00	0.05	0.13	**0.53**	-0.00	-0.26
**G4**	-0.04	-0.02	0.07	0.00	-0.03	-0.01	-0.03	**0.73**	0.02	0.06
**G6**	0.00	0.00	-0.07	-0.02	0.03	-0.00	-0.00	**0.86**	0.04	-0.02

Factor loadings ≥ 0.30 are bolded

## Discussion of the results

This study examined the structural components of the Abilitator, which is a self-report questionnaire on work ability and functioning aimed at the population in a weak labour market position. Our aim was to investigate to what extent the Abilitator has reflective elements in its five reported summary scales, and could structural validity be reached. The data used in the study was collected from the ESF Priority 5 projects, the aim of which is to support work ability, functioning and the overall well-being of those in a weak labour market position. The data corresponded to our prior expectations that the participants would belong to the target group; 64% were long-term unemployed, 63% had at least some health problems and 62% had problems functioning in everyday life. In addition, 72% perceived their work ability as fairly poor or poor. Therefore, the analyses in this study were conducted using the data sample from the intended population.

The models for CFA were specified based on two hypotheses: 1) All the concepts of the Abilitator can be analysed in the same model, and 2) All the concepts can be analysed as separate models. Both of these hypotheses were tested, and the results were compared to the COSMIN cut-off limits for the good model fit and measurement properties of CTT based instruments. The aim was to reach at least an approximate model fit i.e., that the analysed fit indices would fulfil at least acceptable or good criteria, but that in the χ^2^-test the p-value would not need to be over 0.05. It must also be noted that the χ^2^-test easily rejects models with samples larger than 500–600 [45]. The first model that tested all concepts in one model had reasonably high factor loadings of over 0.5 or 0.7, apart from the variable E4 in the “E. Everyday life” and “F. Skills” concepts. There were also high positive correlations among all the other concepts, except for “G. Body”. The SRMR was the only fit index to reach an acceptable value. This first model was too complex and did not have a sufficiently good fit with CFA.

The results for CFA were better with the second approach, in which we tested the Abilitator’s concepts as separate models with partial modifications. All the tested models had high factor loadings of over 0.5 or 0.7, except for item E4 in the “E. Everyday life” and “F. Skills” concepts. The tested second-order factor model for the “C. Inclusion” concept had high factor loadings and reached an approximate fit. With concept “D. Mind” the first-order factor model with letting one pair of error terms to correlate based on the MIs also reached an approximate fit. Similarly, for the”F. Skills” concept, the second-order factor model also resulted in an approximate fit. For this concept we also tested an alternative model without item E4, which is usually counted in its summary scale. This new model reached a very good fit, which means that the concept “F. Skills” could also be used separately without item E4.

The concept “E. Everyday life” with the first-order factor model did not reach an approximate fit even after allowing three error terms to correlate on the basis of the MIs. On the one hand, the result indicates that this concept might need remodelling. On the other hand, this concept might also be formative in nature and therefore the CFA results can be ignored. The concept “C. Everyday life” is a combination of different aspects that are all related to coping with everyday activities, but they do not all need to be present at the same time for a person to find daily coping challenging. Therefore, we can assume that this concept might be based on a formative model. The model that tested the concept “G. Body” was saturated. This means that the model estimated as many parameters as there were pieces of information in the data and therefore the fit was perfect. In this case CFA did not produce a clear result. However, we were able to observe the factor loadings for this model and found that they were either acceptable or high.

The EFA showed that a well-fitting model of the Abilitator had ten different latent dimensions. We made some interesting observations when examining the factor loadings of the Abilitator’s original summary scale items, i.e., which items were highly correlated and clustered in one factor. First, it was evident that some of the items in the two summary scales had similar contents. This was the case with item D1 “I’ve been feeling positive about the future” which clustered in the same factor with F5, F6 and F7 which also contained statements about the future, but from a slightly different perspective. The same was true for item C6 “I am in charge of the course of my life” which clustered with some parts of the original summary scale “C. Inclusion” but also with D4, D5, D7, and D8 which contained aspects of dealing with problems and life control. Second, it was interesting to notice that item F8 “I am able to verbally express myself in different situations” loaded on both the original summary scale “F. Skills” and on C15, C16, and C17, which contained aspects of socializing and friendships. The ability to express oneself verbally was connected to social relationships. Third, psychological well-being seems to be a comprehensive element of work ability and functioning in our study population, as the items in summary scale “D. Mind” clustered with many parts of “C. Inclusion” i.e., social inclusion, social relationships but also with some parts of skills “F. Skills” i.e., cognitive functioning and hopes for the future. This is supported by the literature as we know that unemployment impairs mental health and well-being [1, 51]. Mental disorders, neurological disabilities and substance abuse challenges have also been associated with low work ability in the unemployed [7, 52]. In contrast, good social relationships and experiencing life as meaningful support maintaining work ability in unemployment [53].

The result of EFA can also be viewed from the perspective of both the multidimensional model of work ability [22, 23] and the biopsychosocial model of functioning [24, 25], on which the Abilitator’s underlying theory is based on [29]. In our previous study [29] the items of the Abilitator were linked to the World Health Organization’s International Classification of Functioning, Disability and Health (ICF), which provides a framework to describe and organize information on health-related states [24]. ICF is based on the biopsychosocial model, which provides a holistic understanding of functioning as interactions between different perspectives of health conditions i.e., diseases, disorders, and injuries, and contextual factors i.e., external environmental factors such as climate or social attitudes, and internal personal factors such as age or education [54]. ICF further classifies three levels of functioning: 1) at the level of body or body part, 2) the whole person, and 3) the whole person in a social context. All these levels are interconnected and therefore each disability can be associated with impairments, activity limitations, and restrictions in participation in day-to-day life [54]. In this study, from the Abilitator’s ICF codes’ point of view [29], the connection between the ability to express oneself verbally (d350 conversation) and socializing and friendships (e.g., d750 informal social relationships) can be seen as a logical result as verbal communication is usually an essential a part of social relationships. In addition, the clustering of psychological well-being (e.g., b152 emotional functions) with not just social inclusion (e.g., d7101 appreciation in relationships) and social relationships (e.g., d7500 informal relationships) but also with cognitive functioning (e.g., d160 focusing attention) and future hopes (b1265 optimism) supports the theory of ICF that a human being is a holistic entity [54].

### Significance of the results

This study revealed that the Abilitator is more a combination of separate sets of questions on different aspects of work ability and functioning than one unified questionnaire. The “C. Inclusion”, “D. Mind”, and “F. Skills” concepts had acceptable structural validity [35]. The concept “G. Body” could also be considered reflective, even though the tested model was saturated. The concept “E. Everyday life” was formative in nature and reaching structural validity was thus not relevant. From a practical point of view, this study supports the possibility to utilize the different sections of the Abilitator also separately. In addition, as the section “E. Everyday life” was formative, the responses to each item in this section should be viewed separately as the result of this summary scale does not necessarily reflect different aspects that might be challenging for the respondents in their daily life.

The results of this study support the basic idea of the development of the Abilitator in that the items in its concepts were based more on a theory and their usefulness in practice than on a data-driven approach, in which the items are selected to reflect a single well-defined latent trait [29]. Therefore, each concept of the Abilitator can be interpreted as a sensible, meaningful combination of items of wide-ranging conceptual categories, derived from input from both the professionals and those in weak labour market position. This approach is also supported by Coulter [13], who recommends considering what really matters to the respondents when developing PROMs.

This study also revealed opportunities to further modify the internal structure of the Abilitator. For example, the questionnaire could be made shorter and therefore easier for the respondents to fill in and for the service professionals to interpret. Item reduction could be conducted to form a shorter PROM that includes only those items that reflected the Abilitator’s dimensions the strongest. However, this further development would need to be carried out carefully, without jeopardizing the relevance of the questionnaire to the respondents and its reflection of their situation from different perspectives. An example of such is item “E4. Using the internet, searching for information”, which did not reflect substantially either the section “E. Everyday life” or “F. Skills” even though the item counts as a part of both scales. However, difficulties with digital skills can hinder functioning in contemporary society, including in the labour market and employment.

### Strengths and limitations

The main strength of this study was the carefully conducted examination of the structure of the Abilitator and the structural validation process, which was based on the widely accepted COSMIN methodology [31, 35]. Our study design followed each step of the recommended process for CTT based PROMs of which the underlying theory and the dimensions of the instrument were predetermined. We applied both CFA and EFA to examine the Abilitator’s structure and analysed the results based on COSMIN’s criteria for good measurement properties. At the end of the analysis, we viewed the COSMIN bias checklist for structural validity studies [35]. We confirmed that the sample size was adequate i.e., seven times the number of items and n≥100. We have described the study design and statistical methods used in sufficient detail. The COSMIN guidelines also encourage to conduct internal consistency analysis alongside the structural validity examination phase [35]. We had already successfully implemented the internal consistency analysis with the same items and sections in our previous study on the Abilitator’s test-retest reliability [33]. However, the sample in that study was partly different containing also groups of unemployed individuals slightly closer to the labour market threshold. In the future, we could conduct the internal consistency analysis again with the data sample used in the present study.

However, there are also limitations. The main limitation of this study related to the context of the target population. The study was conducted only with specific data sample collected in Finland and with individuals responding to the Finnish version of the Abilitator. Therefore, the results of this study apply only to the Finnish context. Moreover, the population in the weak labour market position is very heterogenous. In the future, we could repeat the procedures of this study with other subgroups of the unemployed and those responding to the Abilitator in other languages. In addition, those who regularly use an aid when moving around could be studied as a separate subgroup. However, the sample size has not yet been large enough to conduct these subgroup analyses to reach the COSMIN criteria.

## Conclusions

For a co-developed multidimensional self-report questionnaire such as the Abilitator, it might be difficult to reach approximate model fit in one unified model, but this is possible when the questionnaire’s different concepts are analysed separately. As hypothesized, the Abilitator had both reflective and formative elements. Structural validity was reached in the separate concepts that were based on a reflective model. If the Abilitator is revised, shortening the questionnaire could be beneficial for its quicker implementation and interpretation. This study provided valuable information for the possible item reduction to be conducted in the future.

## Supporting information

S1 Appendix The Abilitator questionnaire. (PDF)
