# Supplementary material for: Examination of the structural components of the Abilitator–A self-report questionnaire on work ability and functioning aimed at the population in a weak labour market position
Source: PLoS One. 2024 Mar 19;19(3):e0300182. doi: 10.1371/journal.pone.0300182 (PMC10950251; doi:10.1371/journal.pone.0300182)
Supplement: Attachment — Submitted filename: Response to Reviewers.docx [file pone.0300182.s002.docx]

Dear Prof. Leona Cilar Budler,

We were pleased that you invited us to revise our manuscript (PONE-D-23-26849). We feel that the comments that were provided by the two reviewers dealt with important issues, leading us to revise and improve the manuscript in accordance with those comments. We have also done our best to ensure that our manuscript meets PLOS ONE’s style requirements.

We are also pleased to inform you that we have downloaded an anonymized dataset (n=4555) to the DRYAD data repository <https://doi.org/10.5061/dryad.m905qfv86>. The link to the data will be activated when the article is published. The data does not include the variable for the identification of specific ESF projects, which is used for clustering the participants in the analyses of the present article. In addition, the data does not include any demographic information such as the participants’ age, gender, or duration of unemployment. These particular pieces of information were considered to compromise the anonymity of the participants in the review process of the data sharing protocol conducted by the Finnish Institute of Occupational Health (FIOH). Therefore, the analyses of this paper cannot be fully replicated with the open data. In questions concerning data availability from FIOH more information is available at [Data permits for datasets of the Finnish Institute of Occupational Health | Finnish Institute of Occupational Health (ttl.fi)](https://www.ttl.fi/en/privacy-notice/data-permits-for-datasets-of-the-finnish-institute-of-occupational-health)

Please see below our point-by-point response to the concerns of the reviewers. A marked copy and a clean version of our revised manuscript have also been provided. Page and line numbers refer to a clean copy.

We hope that our response adequately addresses all the points raised and we look forward to hearing from you.

Sincerely,

Mrs. Miia Wikström, on behalf of all the authors

Academic Editor’s Comments to Author:

After careful consideration, we feel that it has merit but does not fully meet PLOS ONE’s publication criteria as it currently stands. Therefore, we invite you to submit a revised version of the manuscript that addresses the points raised during the review process.

**Response 1: Thank you for these encouraging comments. We have tried our best to revise the paper according to the Reviewers’ points.**

Reviewers’ Comments to Author:

Reviewer: 1

Thank you for providing a chance to review this manuscript.

**Response 1: Thank you for your review and very helpful comments.**

**Abstract**
You’d better dividing the paragraph into “Objective”, “Method”, “Results”, and “Conclusion” four parts. And bold these words. This will make it clearer to the reader. Provide a concise summary of the work done and the results obtained as much as possible.

**Response 2: Thank you for these useful suggestions. We have now made these changes to Abstract (page 2 lines 24-45, page 3 lines 46-50)**

**Abstract**

**Objectives**

According to the Consensus-based Standards for the selection of health Measurement Instruments (COSMIN) panel, structural validity describes how well Patient-Reported Outcome Measures’ (PROM) scores reflect the dimensions of the measured construct. Reaching structural validity is important for PROMs that reflect unidimensional effect indicators, but not for formative PROMs in which the items are not necessarily correlated. The main purpose of this study was to examine the structural components of the Abilitator, a co-developed self-report questionnaire on work ability and functioning for the population in a weak labour market position.

**Methods**

We examined to what extent the Abilitator has reflective and formative elements in its five summary scales: “C. Inclusion”, “D. Mind”, “E. Everyday life”, “F. Skills”, and “G. Body”. The Abilitator data sample (n=4555, men 51%, mean age 37 years) was collected in 2017–2022 by the Finnish Institute of Occupational Health in cooperation with the European Social Fund Priority 5 projects in which the participants have multiple challenges to gain employment. For the structural components and validity analysis we implemented both Confirmatory Factor Analysis (CFA) and Exploratory Factor Analysis (EFA).

**Results**

Based on the COSMIN criteria for structural validity, the Abilitator reached approximate model fit with CFA when we analysed the different concepts of the questionnaire separately rather than in one unified model. An exception was “E. Everyday life” which was a formative summary scale, and it did not reach approximate fit. EFA showed that the items in the Abilitator’s summary scales loaded on ten factors.

**Conclusions**

The Abilitator had both reflective and formative elements in its structure. It reached structural validity in those separate concepts that were based on a reflective model. This study revealed interesting connections between different aspects of the Abilitator and produced valuable information for further modification of the questionnaire.

**Materials and Methods
The Abilitator self-report questionnaire**
Line 171-173, Page 7: Please briefly describe in language the differences in meaning represented by changes in scores.

**Response 3: We have added a description according to your suggestion (page7, lines 167-171 and page 8, lines 172-186) and also added Table 1 (page 8) to clarify how the Abilitator’s scores (0-100%) are interpreted.**

The Abilitator contains 84 items under nine domains (sections): “A. Personal details”, “B. Well-being”, “C. Inclusion”, “D. Mind”, “E. Everyday life”, “F. Skills”, “G. Body”, “H. Background information”, and “I. Work and the Future” [29,32,33]. Each section contains 4–14 items (S1 Appendix). The measure of each of the five reported sections C, D, E, F, and G is a summary scale, transformed into a score of 0% to 100% of the selected items (Fig 1, Table 1). The smallest detectable changes in the Abilitator’s summary scale scores are presented in Wikström et al. [33]. The qualitative data is gathered from all the questions in sections A, H, I, and in separate questions of C9-C13, F4, G2, G3, G8, G9-G12. These questions are valuable for the respondents to reflect on, but they also provide useful additional information on the respondent’s situation for the service professional.

In this study, we examined the structural components of the summary scales formed from sections C, D, E, F, and G, which contain the dimensions of social functioning and participation, psychological functioning, managing in everyday life, cognitive functioning and basic skills, and physical functioning, respectively. The “Overall situation” score, which is the mean of the sums obtained from these five summary scales, was not included in the analysis. There are no limit values for this score and the obtained data is used by service professionals to monitor the overall change in the respondent’s work ability and functioning, for example, when the Abilitator is applied for the second time after an intervention. Also, sections A, H, and I were excluded from the analysis since they were developed to provide background information and do not form a summary scale. Even though the Abilitator is available in 10 different languages, in this study we only examined the original Finnish version.

**Table 1. Interpretation of the Abilitator’s results based on the summary scales included in the “Overall situation” score [30].**

|  | **Poor (%)** | **Fairly poor (%)** | **Fairly good (%)** | **Good (%)** |
| --- | --- | --- | --- | --- |
| **C. Inclusion** | 0-23 | 25-48 | 50-73 | 75-100 |
| **D. Mind** | 0-22 | 25-56 | 58-70 | 71-100 |
| **E. Everyday life** | 0-23 | 25-48 | 50-73 | 75-100 |
| **F. Skills** | 0-48 | 50-60 | 61-73 | 75-100 |
| **G. Body** | 0-30 | 40-60 | 61-80 | 90-100 |

These sections are numerically valued on a scale of 0-100%

**We have also described the study population in terms of these Abilitator’s score categories (page 13, lines 301-303).**

In terms of the Abilitator’s score categories (Table 1), 23% of the participants had a poor or fairly poor situation in section “C. Inclusion”, 42% in “D. Mind”, 7% in “E. Everyday life”, 37% in "F. Skills”, and 37% in "G. Body”, respectively.

**The smallest detectable changes in the Abilitator’s summary scale scores are presented in: Wikström et al (2021) “ Intrarater reliability of the Abilitator—a self-report questionnaire on work ability and functioning aimed at the population in a weak labour market position: a test–retest study” BMJ Open.** [**http://dx.doi.org/10.1136/bmjopen-2021-057295**](http://dx.doi.org/10.1136/bmjopen-2021-057295)

**Study sample**
Line 179-188, Page 8: Please add participants' selection methods, inclusion criteria and exclusion criteria.

**Response 4: We have clarified this part according to your suggestion (page 9, lines 195-210).**

The entire Abilitator data (n=54464) has been collected in various services and projects aimed mainly for the unemployed. To ensure that the participants in this study were in a weak labour market position, the sample utilized in this study (n=4555) was collected by FIOH in cooperation only with the European Social Fund Priority 5 projects in 01.10.2017–31.12.2022. Each project (n=150) gave written consent to FIOH to use their Abilitator data in an anonymous format in its research. All the Abilitator respondents in the data were participants in the ESF Priority 5 projects, which aim to improve the work ability and functioning of people not in employment to help them proceed on employment paths and to strengthen social inclusion [40]. In each project the participants took part in activities such as cooking, gardening, social interaction, light physical activity, and rehabilitative work tasks. All the respondents of the Abilitator were adults and had been given written information in advance about the Abilitator’s purpose, data security, the possible later research use of the data in anonymous format, and the voluntary nature of answering the questionnaire. They had also all consented to this by responding to the Abilitator questionnaire. The respondents could choose whether to answer the questionnaire online via the Abilitator online service or in a paper format. In the latter case, the staff of each ESF project transferred the answers to the Abilitator online service with the permission of the respondent.

**In addition, further description of inclusion and exclusion criteria is provided in the Data analysis section (page 10, lines 232-236).**

The Abilitator data we used (n=4555) consisted solely of the data collected from the ESF Priority 5 projects that had two or more participants. This included the individuals who had responded to the Abilitator in Finnish and were between 18 and 74 years old i.e., belonged to the labour force in Finland. The respondents who regularly used an aid when moving around were excluded.

**Methods**

Line 191-203, Page 8: What are qualitative data? Which obey normality and which do not obey? Please provide a detailed explanation. Is there any missing data? If not, please explain how to carry out quality control, etc. If so, please explain how to handle it, such as simple imputation or multiple imputation?

**Response 5: Thank you for your questions. We have divided our response into three parts:**

1) **What are qualitative data?**

**The qualitative data is gathered from all the questions in sections A. Personal details, H. Background information, and I. Work and the Future. In addition, separate questions C9-C13, F4, G2, G3, G8, G9-G12 are qualitative. These questions are valuable for the respondents to reflect on, but they also provide useful additional information on the respondent’s situation for the service professional. We have added this description to text (page 8, lines 172-175).**

The qualitative data is gathered from all the questions in sections A, H, I, and in separate questions of C9-C13, F4, G2, G3, G8, G9-G12. These questions are valuable for the respondents to reflect on, but they also provide useful additional information on the respondent’s situation for the service professional.

**The development of the Abilitator is described in detail in: Wikström et al. (2020) “Development and content validity of the Abilitator: a self-report questionnaire on work ability and functioning aimed at the population in a weak labour market position” BMC Public Health*.*** [**https://doi.org/10.1186/s12889-020-8391-8**](https://doi.org/10.1186/s12889-020-8391-8)

2) **Which obey normality and which do not obey? Please provide a detailed explanation.**

**As we use MLR estimation, which is robust to non-normality, the skewness for continuous data is handled when there is no floor or ceiling effects present. We have mentioned this in the Results and Discussion section (page 14, lines 311-314 )**

No floor or ceiling effects were found, as less than 15% of the responses per analysed item or summary scale scored at the lowest or highest score of the scale. Therefore, the data was considered continuous and normally distributed.

3) **Is there any missing data? If not, please explain how to carry out quality control, etc. If so, please explain how to handle it, such as simple imputation or multiple imputation?**

**Yes, there was missing data (page 14, lines 309-311)**

The percentages of missing values were 0.92% for the whole sample, 0.76% for “C. Inclusion, 1.81% for “D. Mind”, 0.73% for “E. Everyday life”, 0.52% for “F. Skills”, and 0.45% for “G. Body”.

**Missing data was handled with the Missing data at random (MAR) which is a default option in Mplus (page 11, lines 248-249).**

Missing values were handled with the Missing data at random (MAR) option in Mplus.[46].

**MAR is the same as Full information maximum likelihood (FIML) in which includes all the missing data in the analysis. Therefore, we used simple imputation.**

Regarding the unclear order of the use of exploratory factor analysis and confirmatory factor analysis, please add details and explain the reasons.

**Response 6: Thank you for requesting a clarification on this issue. CFA was conducted first and then EFA. We have added a sentence to make this order apparent (page 10, lines 224-225).**

After CFA it was also relevant to conduct EFA to discover how the Abilitator’s items loaded freely.

**The main focus of this study was to conduct a CFA in in accordance with the COSMIN criteria for PROMS of which the dimensions are predetermined. When developing the Abilitator our aim was not to create a data driven PROM based on a unidimensional reflective model, where items are selected based on EFA and then tested in a separate sample with CFA. The Abilitator was developed on the basis of an expert panel-based, content driven model. Our primary hypothesis assumes that this co-developed content is relevant to the target group. EFA has been utilized to examine whether there are same underlying themes between the different domains, of which it is helpful to be aware of. In this kind of self-report questionnaire, a good enough structure is a satisfactory result.**

**More information is provided in our earlier paper Wikström et al. (2020) “Development and content validity of the Abilitator: a self-report questionnaire on work ability and functioning aimed at the population in a weak labour market position” BMC Public Health*.*** [**https://doi.org/10.1186/s12889-020-8391-8**](https://doi.org/10.1186/s12889-020-8391-8)

Also, are the samples for confirmatory factor analysis and exploratory factor analysis the same population? If so, why not separate? Please explain.

**Response 7: CFA and EFA are conducted in the same data. We have added a sentence to clarify this (page 10, line 226-228).**

The same data sample was used for all the analyses, because EFA was not used to develop a reflective, unidimensional, data driven model, which should be confirmed in a separate sample.

**In this study the same data sample could be utilized, because EFA was not used to develop a reflective, unidimensional, data driven model and then utilized to confirm it. Different data samples for EFA and CFA would be relevant if this described unidimensional model was being formed in one sample and confirmed in another.**

**Methods**

Page 8-10: Please add a data analysis section and explain the data analysis software used, as well as detailed data analysis methods or steps. It is best to separate research design from data analysis.

**Response 8: Thank you for this suggestion. We have now divided the Methods section into Research design, Data analysis and an additional Ethics statement section, which was a request from PLOS ONE (pages 9-12, lines 212-290)**.

**We have also added descriptions on the data analysis**

**(page 11, lines 247-251 and 262-263)**

Both CFA and later EFA were conducted with multilevel modeling which considers the clustering of participants in specific ESF projects (TYPE=COMPLEX). Missing values were handled with the Missing data at random (MAR) option in Mplus. [46]. For EFA the type of rotation used was GEOMIN i.e., oblique rotation where the dimensions of the Abilitator were allowed to correlate [45, 46].

In the estimation phase, we estimated the factor loadings and residual error terms for all the models using the MLR method (ESTIMATOR = MLR).

**(page 12, lines 283-284)**

For CFA we used STYDYX standardization which is recommended for continuous variables [46].

**The software used i.e., Mplus is described in detail in reference 46. Muthén L, Muthén B. In Mplus user's guide (8 ed.). Los Angeles, CA, USA: Author. Available from:** [**http://www.statmodel.com/**](http://www.statmodel.com/)

**Results and discussion**
**Study population characteristics**
Table 1, Page 12: If there are no special requirements for the magazine, please use a three-line form.

**Response 9: The former Table 1, now Table 2 has been modified in line with PLOS ONE’s style requirements (page 13).**

Line 266, Page 12: Is this a note for the table? Please pay attention to the formatting details, don't you even have a corner marker.

**Response 10: Yes, those were footnotes for Table 2. Unfortunately, the lines moved to next page when the pdf of the manuscript was formed. We hope this issue doesn’t occur again. We have also decreased the font size of the foot notes to make them clearer.**

**Confirmatory factor analysis**
Table 2, Page 13: If there are no special requirements for the magazine, please use a three-line form.

**Response 11: The former Table 2, now Table 3 has been modified in line with PLOS ONE’s style requirements (page 15). In addition, the heading of this whole section has been renamed to Confirmatory and Exploratory factor analysis (page 14, line 308)**

Table 3, Page 14: This icon is not aesthetically pleasing, please beautify your results.

**Response 12: We were considering very hard how to make Table 4 (formerly Table 3), page 15 more aesthetically pleasing without losing any of its content but were able to make only very small modifications. We are happy to take further suggestions about how to make the table look better.**

Figures: The clarity of the images is relatively low. Please pay attention to improving your resolution according to the magazine's requirements.

**Response 13: This is true. These images turned out blurry when the original pdf files were transformed to the format required by PLOS ONE. If this problem persists, we will do our best to sort it in cooperation between the experts of PLOS ONE and our organization by the time of publication.**

**Discussion of the results**

Page 17: Is it supported by the literature?

**Response 14: Thank you for requesting this information. We have added a few sentences and a separate paragraph to expand on this issue (page 19-20, lines 408-432)**

This is supported by the literature as we know that unemployment impairs mental health and well-being [1, 51]. Mental disorders, neurological disabilities and substance abuse challenges have also been associated with low work ability in the unemployed [7,52]. In contrast, good social relationships and experiencing life as meaningful support maintaining work ability in unemployment [53].

The result of EFA can also be viewed from the perspective of both the multidimensional model of work ability [22,23] and the biopsychosocial model of functioning [24,25], on which the Abilitator’s underlying theory is based on [29]. In our previous study [29] the items of the Abilitator were linked to the World Health Organization’s International Classification of Functioning, Disability and Health (ICF), which provides a framework to describe and organize information on health-related states [24]. ICF is based on the biopsychosocial model, which provides a holistic understanding of functioning as interactions between different perspectives of health conditions i.e., diseases, disorders, and injuries, and contextual factors i.e., external environmental factors such as climate or social attitudes, and internal personal factors such as age or education [54]. ICF further classifies three levels of functioning: 1) at the level of body or body part; 2) the whole person; and 3) the whole person in a social context. All these levels are interconnected and therefore each disability can be associated with impairments, activity limitations, and restrictions in participation in day-to-day life. [54]. In this study, from the Abilitator’s ICF codes’ point of view [29], the connection between the ability to express oneself verbally (d350 conversation) and socializing and friendships (e.g., d750 informal social relationships) can be seen as a logical result as verbal communication is usually an essential a part of social relationships. In addition, the clustering of psychological well-being (e.g., b152 emotional functions) with not just social inclusion (e.g., d7101 appreciation in relationships) and social relationships (e.g., d7500 informal relationships) but also with cognitive functioning (e.g., d160 focusing attention) and future hopes (b1265 optimism) supports the theory of ICF that a human being is a holistic entity [54].

**Significance of the results**
Page 17: Please increase the discussion on the limitations of this study, including potential biases and sources of uncertainty, and provide a reflection on this study.

**Response 15: We have expanded on this with a subsection titled “Strengths and limitations” (pages 21-22, lines 467-492)**

**Strengths and limitations**

The main strength of the study was the well-structured examination of the structure of the Abilitator and the structural validation process, which was based on the widely accepted COSMIN methodology [31,35]. Our study design followed each step of the recommended process for CTT based PROMs of which the underlying theory and the dimensions of the instrument were predetermined. We applied both CFA and EFA to examine the Abilitator’s structure and analysed the results based on COSMIN’s criteria for good measurement properties. At the end of the analysis, we viewed the COSMIN bias checklist for structural validity studies [35]. We confirmed that the sample size was adequate i.e., seven times the number of items and n≥100. We have described the study design and statistical methods used in sufficient detail. The COSMIN guidelines also encourage to conduct internal consistency analysis alongside the structural validity examination phase [35]. We had already successfully implemented the internal consistency analysis with the same items and sections in our previous study on the Abilitator’s test-retest reliability [33]. However, the sample in that study was partly different containing also groups of unemployed individuals slightly closer to the labour market threshold. In the future, we could conduct the internal consistency analysis again with the data sample used in the present study.

However, there are also limitations. The main limitation of this study related to the context of the target population. The study was conducted only with specific data sample collected in Finland and with individuals responding to the Finnish version of the Abilitator. Therefore, the results of this study apply only to the Finnish context. Moreover, the group of the population in the weak labour market position is very heterogenous. In the future, we could repeat the procedures of this study with other subgroups of the unemployed and those responding to the Abilitator in some other language than Finnish. In addition, those who regularly use an aid when moving around could be studied as a separate subgroup. However, the sample size has not yet been large enough to conduct these subgroup analyses to reach the COSMIN criteria.

**We also added discussion on the significance of the results (pages 20-21, lines 434-465).**

**Significance of the results**

This study revealed that the Abilitator is more a combination of separate sets of questions on different aspects of work ability and functioning than one unified questionnaire. The “C. Inclusion”, “D. Mind”, and “F. Skills” concepts can be considered reflective as they reached approximate fit with CFA. Therefore, these concepts had acceptable structural validity according to the COSMIN criteria for PROMs based on CTT [35]. The concept “G. Body” could also be considered reflective, even though the tested model was saturated. The concept “E. Everyday life” was formative in nature and reaching structural validity was thus not relevant. From a practical point of view, this study supports the possibility to utilize the different sections of the Abilitator also as separate PROMs. In addition, as the section “E. Everyday life” was formative, the responses to each item in this section should be viewed separately as the result of this summary scale does not necessarily reflect the separate items that might be challenging for the respondent.

The results of this study support the basic idea of the development of the Abilitator in that the items in its concepts were based more on a theory and their usefulness in practice than on a data-driven approach, in which the items are selected to reflect a single well-defined latent trait [29]. Therefore, each concept of the Abilitator can be interpreted as a sensible, meaningful combination of items of wide-ranging conceptual categories, derived from input from both the professionals and those in weak labour market position. This approach is also supported by Coulter [13], who recommends considering what really matters to the respondents when developing PROMs.

This study also revealed opportunities to further modify the internal structure of the Abilitator. For example, the questionnaire could be made shorter and therefore easier for the respondents to fill in and for the service professionals to interpret. Based on the information from both the CFA and EFA, item reduction could be conducted to form a shorter PROM that includes only those items that reflected the Abilitator’s dimensions the strongest. However, this further development would need to be carried out carefully, without jeopardizing the relevance of the questionnaire to the respondents and its reflection of their situation from different perspectives. An example of such is item “E4. Using the internet, searching for information”, which did not reflect substantially either the section “E. Everyday life” or “F. Skills” even though the item counts as a part of both scales. However, difficulties with digital skills can hinder functioning in contemporary society, including in the labour market and employment. Therefore, it is necessary to consider what information is important to view when employment is promoted.

**Overall**

The results and discussion sections should be separated. The results are only objectively presented based on the analysis data of this time. The logic of this section needs to be strengthened.

**Response 16: We have tried to strengthen the content of the Results and Discussion section as per your above suggestions as well as divided this section to subsections. However, we did not separate these two sections. We have followed the PLOS ONE’s submission guidelines in which combining is one of the given options for authors to choose:**

**“Results, Discussion, Conclusions. These sections may all be separate or may be combined to create a mixed Results/Discussion section (commonly labeled “Results and Discussion”) or a mixed Discussion/Conclusions section (commonly labeled “Discussion”). These sections may be further divided into subsections, each with a concise subheading, as appropriate. These sections have no word limit, but the language should be clear and concise.”** [Submission Guidelines | PLOS ONE](https://journals.plos.org/plosone/s/submission-guidelines)

**Conclusions**
Page 18: Please explain the practical significance of the research results.

**Response 17: We have added an extra sentence to the Conclusions section (Page 22, lines 494-501) and several sentences to the Significance of the Results section (see Response 15) to explain the practical significance of the results.**

**Conclusions**

For a co-developed multidimensional self-report questionnaire such as the Abilitator, it might be difficult to reach approximate model fit in one unified model, but this is possible when the questionnaire’s different concepts are analysed separately. As hypothesized, the Abilitator had both reflective and formative elements. Structural validity was reached in the separate concepts that were based on a reflective model. If the Abilitator is revised, shortening the questionnaire could be beneficial for its quicker implementation and interpretation. This study provided valuable information for the possible item reduction to be conducted in the future.

Overall, I feel that your writing is not rigorous enough and your logic needs to be strengthened. The biggest issue is the research design and data analysis methods section. I think it's not enough to support publication.

**Response 18: We have revised the content of these sections according to your excellent suggestions (page 9-12, lines 213-284). Our goal was to explain all the methods, phases and results of this study as clearly as possible so that even a person who is not familiar with the subject and these specific statistical analysis methods could understand this relatively complex study. We hope that our revisions have made our manuscript clearer and strong enough to support its publication.**

Reviewer: 2

Ingenious way to calculate structural validity: through CFA with all concepts in the same model and considered separately.

**Response 1: We highly appreciate this encouraging feedback.**

Line 269 - it seems that instead of 136 it is 132.

**Response 2: Thank you very much for noticing our mistake. We have now corrected the value from 136 to 132 (3x44) (page 14, line 310).**

The requirement was either over 400 or 3–15 times the number of the analysed 44 variables i.e., 132 to 660.

Table 3 - I think it would be important that factor loadings to be reproduced numerically.

**Response 2: A good suggestion, thank you. We have now added the numerical factor loadings to Table 4 which was previously Table 3 (page 16).**

**Table 4. Exploratory Factor Analysis with ten factors.**

|  | **F1** | **F2** | **F3** | **F4** | **F5** | **F6** | **F7** | **F8** | **F9** | **F10** |
| --- | --- | --- | --- | --- | --- | --- | --- | --- | --- | --- |
| **C1** | **0.47** | -0.11 | 0.01 | 0.22 | 0.01 | 0.04 | 0.01 | 0.11 | -0.02 | 0.14 |
| **C2** | **0.74** | 0.04 | 0.02 | 0.03 | -0.10 | 0.01 | -0.03 | -0.05 | 0.10 | -0.09 |
| **C3** | **0.61** | 0.01 | -0.02 | 0.16 | 0.12 | -0.10 | -0.00 | 0.06 | 0.04 | 0.13 |
| **C4** | **0.83** | -0.02 | -0.03 | 0.11 | -0.03 | -0.08 | 0.01 | 0.01 | 0.04 | 0.01 |
| **C5** | **0.57** | 0.00 | 0.04 | -0.04 | 0.10 | 0.17 | 0.01 | 0.00 | 0.03 | -0.01 |
| **C6** | **0.33** | -0.00 | 0.13 | 0.05 | **0.32** | 0.12 | -0.03 | 0.06 | -0.12 | 0.06 |
| **C7** | **0.49** | 0.11 | 0.19 | 0.05 | 0.05 | 0.11 | 0.00 | -0.01 | -0.11 | -0.00 |
| **C8** | 0.27 | 0.09 | -0.00 | **0.49** | 0.05 | 0.04 | 0.01 | -0.01 | -0.09 | -0.02 |
| **C14** | 0.10 | 0.02 | 0.04 | **0.58** | 0.00 | -0.01 | 0.07 | -0.02 | 0.03 | 0.04 |
| **C15** | 0.04 | **0.38** | 0.11 | **0.47** | 0.02 | -0.00 | 0.04 | -0.00 | -0.02 | -0.07 |
| **C16** | 0.05 | **0.87** | 0.01 | 0.09 | 0.02 | -0.00 | -0.02 | 0.01 | -0.01 | -0.02 |
| **C17** | -0.03 | **0.84** | 0.03 | 0.02 | -0.03 | 0.00 | 0.01 | -0.00 | 0.04 | 0.07 |
| **D1** | 0.03 | 0.04 | -0.03 | -0.06 | 0.05 | **0.60** | 0.07 | 0.02 | -0.03 | 0.01 |
| **D2** | **0.52** | 0.09 | 0.06 | -0.05 | 0.07 | 0.20 | 0.08 | -0.04 | -0.00 | -0.12 |
| **D3** | 0.20 | 0.01 | 0.01 | 0.03 | 0.19 | 0.15 | 0.29 | 0.06 | -0.22 | -0.02 |
| **D4** | 0.08 | 0.06 | 0.04 | 0.03 | **0.39** | 0.12 | 0.11 | -0.01 | 0.07 | -0.06 |
| **D5** | 0.07 | 0.04 | 0.03 | 0.01 | **0.44** | -0.01 | **0.32** | -0.02 | -0.01 | 0.01 |
| **D6** | 0.23 | 0.12 | -0.08 | 0.30 | 0.11 | 0.14 | 0.02 | -0.02 | 0.07 | -0.04 |
| **D7** | 0.02 | 0.04 | 0.02 | 0.05 | **0.71** | 0.02 | 0.03 | 0.01 | 0.01 | 0.10 |
| **D8** | -0.00 | 0.06 | 0.28 | -0.02 | **0.55** | 0.05 | -0.04 | 0.00 | 0.05 | -0.05 |
| **D9** | 0.20 | -0.02 | 0.01 | 0.12 | 0.28 | 0.24 | 0.12 | 0.00 | -0.00 | -0.02 |
| **E1** | -0.06 | -0.02 | **0.63** | 0.06 | 0.05 | 0.06 | 0.04 | 0.15 | -0.07 | -0.02 |
| **E2** | -0.03 | 0.03 | **0.71** | -0.01 | -0.04 | 0.06 | 0.05 | 0.07 | -0.09 | 0.24 |
| **E3** | 0.04 | 0.08 | **0.67** | -0.08 | 0.06 | -0.03 | 0.06 | -0.06 | 0.03 | **0.33** |
| **E4** | 0.03 | -0.16 | 0.20 | 0.01 | 0.02 | -0.03 | 0.23 | 0.03 | 0.24 | 0.29 |
| **E5** | 0.03 | -0.10 | **0.44** | -0.01 | 0.25 | -0.12 | 0.11 | -0.05 | 0.01 | -0.03 |
| **E6** | 0.05 | -0.00 | **0.53** | 0.05 | 0.14 | 0.00 | 0.04 | 0.15 | -0.02 | -0.26 |
| **E7** | 0.09 | -0.01 | **0.47** | -0.02 | -0.03 | 0.04 | 0.14 | 0.05 | -0.01 | -0.22 |
| **E8** | -0.04 | -0.05 | **0.61** | 0.11 | 0.08 | -0.04 | -0.05 | 0.01 | 0.09 | -0.08 |
| **E9** | 0.09 | 0.03 | **0.63** | -0.01 | 0.07 | -0.02 | -0.08 | -0.07 | 0.09 | 0.01 |
| **E10** | 0.05 | 0.12 | **0.61** | 0.01 | -0.09 | 0.07 | -0.00 | 0.08 | 0.00 | 0.20 |
| **E11** | 0.01 | 0.02 | **0.47** | 0.24 | -0.03 | 0.04 | -0.02 | -0.02 | 0.19 | 0.05 |
| **F1** | -0.05 | 0.01 | 0.18 | 0.07 | 0.09 | 0.01 | 0.60 | -0.07 | -0.00 | 0.01 |
| **F2** | -0.01 | 0.02 | -0.03 | 0.03 | -0.04 | 0.07 | 0.66 | 0.03 | 0.23 | 0.08 |
| **F3** | 0.02 | -0.00 | 0.15 | 0.00 | 0.01 | -0.04 | 0.60 | 0.03 | 0.01 | -0.08 |
| **F5** | 0.27 | -0.01 | 0.09 | 0.01 | 0.00 | **0.61** | 0.03 | 0.00 | 0.02 | 0.00 |
| **F6** | 0.02 | -0.05 | 0.01 | 0.11 | -0.03 | **0.67** | -0.04 | 0.01 | **0.37** | 0.05 |
| **F7** | -0.04 | 0.01 | 0.06 | 0.08 | 0.08 | **0.53** | -0.01 | 0.01 | **0.43** | -0.06 |
| **F8** | 0.13 | 0.15 | -0.02 | -0.04 | -0.01 | 0.23 | 0.17 | 0.04 | **0.38** | -0.04 |
| **F9** | 0.03 | **0.46** | 0.01 | 0.00 | 0.15 | -0.01 | 0.08 | 0.02 | **0.32** | 0.02 |
| **F10** | 0.04 | 0.13 | 0.01 | -0.05 | 0.12 | 0.04 | 0.22 | 0.06 | **0.39** | 0.06 |
| **G1** | 0.09 | 0.05 | 0.13 | -0.00 | 0.00 | 0.05 | 0.13 | **0.53** | -0.00 | -0.26 |
| **G4** | -0.04 | -0.02 | 0.07 | 0.00 | -0.03 | -0.01 | -0.03 | **0.73** | 0.02 | 0.06 |
| **G6** | 0.00 | 0.00 | -0.07 | -0.02 | 0.03 | -0.00 | -0.00 | **0.86** | 0.04 | -0.02 |

Factor loadings ≥ 0.30 are bolded

It does not appear out of nowhere how the questionnaire was completed by the weak labor market position population? Online?

**Response 3: Thank you for bringing this up. We have added the description under the subsection “Study sample” (page 9, lines 194-210 ).**

**Study sample**

The entire Abilitator data (n=54464) has been collected in various services and projects aimed mainly for the unemployed. To ensure that the participants in this study were in a weak labour market position, the sample utilized in this study (n=4555) was collected by FIOH in cooperation only with the European Social Fund Priority 5 projects in 01.10.2017–31.12.2022. Each project (n=150) gave written consent to FIOH to use their Abilitator data in an anonymous format in its research. All the Abilitator respondents in the data were participants in the ESF Priority 5 projects, which aim to improve the work ability and functioning of people not in employment to help them proceed on employment paths and to strengthen social inclusion [40]. In each project the participants took part in activities such as cooking, gardening, social interaction, light physical activity, and rehabilitative work tasks. All the respondents of the Abilitator were adults and had been given written information in advance about the Abilitator’s purpose, data security, the possible later research use of the data in anonymous format, and the voluntary nature of answering the questionnaire. They had also all consented to this by responding to the Abilitator questionnaire. The respondents could choose whether to answer the questionnaire online via the Abilitator online service or in a paper format. In the latter case, the staff of each ESF project transferred the answers to the Abilitator online service with the permission of the respondent.
