# Supplementary material for: Examination of the structural components of the Abilitator–A self-report questionnaire on work ability and functioning aimed at the population in a weak labour market position
Source: PLoS One. 2024 Mar 19;19(3):e0300182. doi: 10.1371/journal.pone.0300182 (PMC10950251; doi:10.1371/journal.pone.0300182)
Supplement: Attachment — Submitted filename: Response to Reviewers.docx [file pone.0300182.s003.docx]

Dear Prof. Leona Cilar Budler,

We were pleased that you invited us to revise our manuscript (PONE-D-23-26849R1). We feel that the further comments that were provided by the Reviewer 1 dealt with important issues, leading us to revise and improve the manuscript in accordance with those comments. We have also reviewed the reference list to ensure it is complete and correct.

Please see below our point-by-point response to the concerns of the reviewer. A marked copy and a clean version of our revised manuscript have also been provided. Page and line numbers refer to a clean copy.

We hope that our response adequately addresses all the points raised and we look forward to hearing from you.

Sincerely,

Mrs. Miia Wikström, on behalf of all the authors

Academic Editor’s Comments to Author:

After careful consideration, we feel that it has merit but does not fully meet PLOS ONE’s publication criteria as it currently stands. Therefore, we invite you to submit a revised version of the manuscript that addresses the points raised during the review process.

**Response 1: Thank you for these encouraging comments. We have tried our best to revise the paper according to the points raised.**

Reviewers’ Comments to Author:

Reviewer: 1

Thank you for providing a chance to review this manuscript.

**Response 1: Thank you for your review and helpful comments.**

Abstract

Objective: There are too many words, please briefly summarize the research background and purpose.

**Response 2: Thank you for this good suggestion. We have now made changes to Objectives (page 2 lines 24-29)**

**Objectives**

According to the Consensus-based Standards for the selection of health Measurement Instruments (COSMIN) panel, structural validity describes how well Patient-Reported Outcome Measures’ (PROM) scores reflect the dimensions of the measured construct. The main purpose of this study was to examine the structural components of the Abilitator, a co-developed self-report questionnaire on work ability and functioning for the population in a weak labour market position.

Materials and Methods
The title is quite confusing, please pay attention to the subordinate relationship between the title and subtitle.

**Response 3: Thank you for this very useful comment. We have tried to follow the PLOS ONE’s submission guidelines regarding the “Materials and Methods” section that the journal requires, see** [**Submission Guidelines | PLOS ONE**](https://journals.plos.org/plosone/s/submission-guidelines). **However, we have made some changes to headings based on your suggestion. First, we unified the formatting of titles so that this article contains only either level 1 headings (Bold type, 18 pt font) for major sections or level 2 headings (Bold type, 16 pt font) for sub-sections of major sections. Second, we deleted the heading “Methods” (marked copy page 9, line 212), which partly duplicated the major heading “Materials and Methods”. We hope these changes address the points you raised.**

The previous review comments were well revised, but there are still a few parts that need to be improved. Please review the expression of the article as much as possible from the reader's perspective.

**Response 4: We highly appreciate your feedback. We have tried our best to review the expression of the article from the reader’s perspective and made some amendments particularly to the Significance of the results section (pages 20-21, lines 433-461, marked copy pages 20-21, lines 437-469) and the Strengths and limitations section (page 21-22, lines 463-488, marked copy pages 21-22, lines 471-496). We also deleted two sentences in the Study sample section (marked copy page 9, lines 204-208) as they repeated the Ethics Statement (page 12, lines 285-289, marked copy pages 12-13, lines 291-295).**

Reviewer: 2

I think the manuscript improved after the changes made by the authors. I consider it a robust piece of research that contributes to the assessment literature by introducing this auxiliary tool necessary in the evaluation of the population in a weak labor market position.

**Response 1: Thank you very much for your review and encouraging feedback.**
