# Supplementary material for: Examination of the structural components of the Abilitator–A self-report questionnaire on work ability and functioning aimed at the population in a weak labour market position
Source: PLoS One. 2024 Mar 19;19(3):e0300182. doi: 10.1371/journal.pone.0300182 (PMC10950251; doi:10.1371/journal.pone.0300182)
Supplement: Peer review history

# Peer review: Decision Letter 0

**DOI:** 10.1371/journal.pone.0300182.r001

**Authors:** Leona Cilar Budler

15 Dec 2023

PONE-D-23-26849Examination of the structural components of the Abilitator – a self-report questionnaire on work ability and functioning aimed at the population in a weak labour market positionPLOS ONE

Dear Dr. Wikström,

Thank you for submitting your manuscript to PLOS ONE. After careful consideration, we feel that it has merit but does not fully meet PLOS ONE’s publication criteria as it currently stands. Therefore, we invite you to submit a revised version of the manuscript that addresses the points raised during the review process.

Please submit your revised manuscript by Jan 29 2024 11:59PM. If you will need more time than this to complete your revisions, please reply to this message or contact the journal office at plosone@plos.org. When you're ready to submit your revision, log on to https://www.editorialmanager.com/pone/ and select the 'Submissions Needing Revision' folder to locate your manuscript file.

Please include the following items when submitting your revised manuscript:

- A rebuttal letter that responds to each point raised by the academic editor and reviewer(s). You should upload this letter as a separate file labeled 'Response to Reviewers'.
- A marked-up copy of your manuscript that highlights changes made to the original version. You should upload this as a separate file labeled 'Revised Manuscript with Track Changes'.
- An unmarked version of your revised paper without tracked changes. You should upload this as a separate file labeled 'Manuscript'.

If you would like to make changes to your financial disclosure, please include your updated statement in your cover letter. Guidelines for resubmitting your figure files are available below the reviewer comments at the end of this letter.

If applicable, we recommend that you deposit your laboratory protocols in protocols.io to enhance the reproducibility of your results. Protocols.io assigns your protocol its own identifier (DOI) so that it can be cited independently in the future. For instructions see: https://journals.plos.org/plosone/s/submission-guidelines#loc-laboratory-protocols. Additionally, PLOS ONE offers an option for publishing peer-reviewed Lab Protocol articles, which describe protocols hosted on protocols.io. Read more information on sharing protocols at https://plos.org/protocols?utm_medium=editorial-email&utm_source=authorletters&utm_campaign=protocols.

We look forward to receiving your revised manuscript.

Kind regards,

Leona Cilar Budler

Academic Editor

PLOS ONE

Journal Requirements:

When submitting your revision, we need you to address these additional requirements.

1. Please ensure that your manuscript meets PLOS ONE's style requirements, including those for file naming. The PLOS ONE style templates can be found at 

https://journals.plos.org/plosone/s/file?id=wjVg/PLOSOne_formatting_sample_main_body.pdf and 

https://journals.plos.org/plosone/s/file?id=ba62/PLOSOne_formatting_sample_title_authors_affiliations.pdf

2. Note from Emily Chenette, Editor in Chief of PLOS ONE, and Iain Hrynaszkiewicz, Director of Open Research Solutions at PLOS: 

Did you know that depositing data in a repository is associated with up to a 25% citation advantage (https://doi.org/10.1371/journal.pone.0230416)? If you’ve not already done so, consider depositing your raw data in a repository to ensure your work is read, appreciated and cited by the largest possible audience. You’ll also earn an Accessible Data icon on your published paper if you deposit your data in any participating repository (https://plos.org/open-science/open-data/#accessible-data).

3. We note that you have indicated that data from this study are available upon request. PLOS only allows data to be available upon request if there are legal or ethical restrictions on sharing data publicly. For more information on unacceptable data access restrictions, please see http://journals.plos.org/plosone/s/data-availability#loc-unacceptable-data-access-restrictions. 

In your revised cover letter, please address the following prompts:

a) If there are ethical or legal restrictions on sharing a de-identified data set, please explain them in detail (e.g., data contain potentially sensitive information, data are owned by a third-party organization, etc.) and who has imposed them (e.g., an ethics committee). Please also provide contact information for a data access committee, ethics committee, or other institutional body to which data requests may be sent.

b) If there are no restrictions, please upload the minimal anonymized data set necessary to replicate your study findings as either Supporting Information files or to a stable, public repository and provide us with the relevant URLs, DOIs, or accession numbers. For a list of acceptable repositories, please see http://journals.plos.org/plosone/s/data-availability#loc-recommended-repositories.

We will update your Data Availability statement on your behalf to reflect the information you provide.

4. Your ethics statement should only appear in the Methods section of your manuscript. If your ethics statement is written in any section besides the Methods, please move it to the Methods section and delete it from any other section. Please ensure that your ethics statement is included in your manuscript, as the ethics statement entered into the online submission form will not be published alongside your manuscript. 

5. Please include captions for your Supporting Information files at the end of your manuscript, and update any in-text citations to match accordingly. Please see our Supporting Information guidelines for more information: http://journals.plos.org/plosone/s/supporting-information. 

**Additional Editor Comments:**

There are major issues pointed out by reviewers. Please read them all carefully and revise paper in order to consider your paper for publication. Also, check all journal guidelines.

[Note: HTML markup is below. Please do not edit.]

Reviewers' comments:

Reviewer's Responses to Questions

**Comments to the Author**

1. Is the manuscript technically sound, and do the data support the conclusions?

The manuscript must describe a technically sound piece of scientific research with data that supports the conclusions. Experiments must have been conducted rigorously, with appropriate controls, replication, and sample sizes. The conclusions must be drawn appropriately based on the data presented. 

Reviewer #1: Partly

Reviewer #2: Yes

**********

2. Has the statistical analysis been performed appropriately and rigorously? 

Reviewer #1: No

Reviewer #2: Yes

**********

3. Have the authors made all data underlying the findings in their manuscript fully available?

The PLOS Data policy requires authors to make all data underlying the findings described in their manuscript fully available without restriction, with rare exception (please refer to the Data Availability Statement in the manuscript PDF file). The data should be provided as part of the manuscript or its supporting information, or deposited to a public repository. For example, in addition to summary statistics, the data points behind means, medians and variance measures should be available. If there are restrictions on publicly sharing data—e.g. participant privacy or use of data from a third party—those must be specified.

Reviewer #1: Yes

Reviewer #2: No

**********

4. Is the manuscript presented in an intelligible fashion and written in standard English?

PLOS ONE does not copyedit accepted manuscripts, so the language in submitted articles must be clear, correct, and unambiguous. Any typographical or grammatical errors should be corrected at revision, so please note any specific errors here.

Reviewer #1: No

Reviewer #2: Yes

**********

5. Review Comments to the Author

Please use the space provided to explain your answers to the questions above. You may also include additional comments for the author, including concerns about dual publication, research ethics, or publication ethics. (Please upload your review as an attachment if it exceeds 20,000 characters)

Reviewer #1: ID: PONE-D-23-26849

Title: Examination of the structural components of the Abilitator – a self-report questionnaire on work ability and functioning aimed at the population in a weak labour market position

Thank you for providing a chance to review this manuscript.

Detailed information:

Abstract

You'd better dividing the paragraph into “Objective”, “Method”, “Results”, and “Conclusion” four parts. And bold these words. This will make it clearer to the reader. Provide a concise summary of the work done and the results obtained as much as possible.

Materials and Methods

The Abilitator self-report questionnaire

Line 171-173, Page 7: Please briefly describe in language the differences in meaning represented by changes in scores.

Study sample

Line 179-188, Page 8: Please add participants' selection methods, inclusion criteria and exclusion criteria.

Methods

Line191-203, Page 8: 1) What are measurement data? What are qualitative data? Which obey normality and which do not obey? Please provide a detailed explanation. 2) Is there any missing data? If not, please explain how to carry out quality control, etc. If so, please explain how to handle it, such as simple imputation or multiple imputation? 3) Regarding the unclear order of the use of exploratory factor analysis and confirmatory factor analysis, please add details and explain the reasons. 4) Also, are the samples for confirmatory factor analysis and exploratory factor analysis the same population? If so, why not separate? Please explain.

Page 8-10: Please add a data analysis section and explain the data analysis software used, as well as detailed data analysis methods or steps. It is best to separate research design from data analysis.

Results and discussion

Study population characteristics

Table 1, Page 12: If there are no special requirements for the magazine, please use a three-line form.

Line 266, Page 12: Is this a note for the table? Please pay attention to the formatting details, don't you even have a corner marker?

Confirmatory factor analysis

Table 2, Page 13: If there are no special requirements for the magazine, please use a three-line form.

Table 3, Page 14: This icon is not aesthetically pleasing, please beautify your results.

Figures: The clarity of the images is relatively low. Please pay attention to improving your resolution according to the magazine's requirements.

Discussion of the results

Page 17: Is it supported by the literature?

Significance of the results

Page 17: Please increase the discussion on the limitations of this study, including potential biases and sources of uncertainty, and provide a reflection on this study.

Overall: The results and discussion sections should be separated. The results are only objectively presented based on the analysis data of this time. The logic of this section needs to be strengthened.

Conclusions

Page 18: Please explain the practical significance of the research results.

Overall, I feel that your writing is not rigorous enough and your logic needs to be strengthened. The biggest issue is the research design and data analysis methods section. I think it's not enough to support publication.

Thank you and my best,

Your reviewer

Reviewer #2: Ingenious way to calculate structural validity: through CFA with all concepts in the same model and considered separately.

Line 269 - it seems that instead of 136 it is 132.

Table 3 - I think it would be important that factor loadings to be reproduced numerically.

It does not appear out of nowhere how the questionnaire was completed by the weak labor market position population? Online?

**********

6. PLOS authors have the option to publish the peer review history of their article (what does this mean?). If published, this will include your full peer review and any attached files.

If you choose “no”, your identity will remain anonymous but your review may still be made public.

**Do you want your identity to be public for this peer review?** For information about this choice, including consent withdrawal, please see our Privacy Policy.

Reviewer #1: No

Reviewer #2: No

**********

[NOTE: If reviewer comments were submitted as an attachment file, they will be attached to this email and accessible via the submission site. Please log into your account, locate the manuscript record, and check for the action link "View Attachments". If this link does not appear, there are no attachment files.]

While revising your submission, please upload your figure files to the Preflight Analysis and Conversion Engine (PACE) digital diagnostic tool, https://pacev2.apexcovantage.com/. PACE helps ensure that figures meet PLOS requirements. To use PACE, you must first register as a user. Registration is free. Then, login and navigate to the UPLOAD tab, where you will find detailed instructions on how to use the tool. If you encounter any issues or have any questions when using PACE, please email PLOS at figures@plos.org. Please note that Supporting Information files do not need this step.

# Author comment: Author response to Decision Letter 0

**DOI:** 10.1371/journal.pone.0300182.r002

31 Jan 2024

Academic Editor’s Comments to Author:

After careful consideration, we feel that it has merit but does not fully meet PLOS ONE’s publication criteria as it currently stands. Therefore, we invite you to submit a revised version of the manuscript that addresses the points raised during the review process.

Response 1: Thank you for these encouraging comments. We have tried our best to revise the paper according to the Reviewers’ points. 

Reviewers’ Comments to Author:

The page and line numbers refer to clean copy of the manuscript. 

Reviewer: 1

Thank you for providing a chance to review this manuscript.

Response 1: Thank you for your review and very helpful comments.

Abstract

You’d better dividing the paragraph into “Objective”, “Method”, “Results”, and “Conclusion” four parts. And bold these words. This will make it clearer to the reader. Provide a concise summary of the work done and the results obtained as much as possible.

Response 2: Thank you for these useful suggestions. We have now made these changes to Abstract (page 2 lines 24-45, page 3 lines 46-50) 

Materials and Methods

The Abilitator self-report questionnaire

Line 171-173, Page 7: Please briefly describe in language the differences in meaning represented by changes in scores.

Response 3: We have added a description according to your suggestion (page7, lines 167-171 and page 8, lines 172-186) and also added Table 1 (page 8) to clarify how the Abilitator’s scores (0-100%) are interpreted. We have also described the study population in terms of these Abilitator’s score categories (page 13, lines 301-303). The smallest detectable changes in the Abilitator’s summary scale scores are presented in: Wikström et al. (2021) “Intrarater reliability of the Abilitator—a self-report questionnaire on work ability and functioning aimed at the population in a weak labour market position: a test–retest study” BMJ Open. http://dx.doi.org/10.1136/bmjopen-2021-057295

Study sample

Line 179-188, Page 8: Please add participants' selection methods, inclusion criteria and exclusion criteria. 

Response 4: We have clarified this part according to your suggestion (page 9, lines 195-210). 

In addition, further description of inclusion and exclusion criteria is provided in the Data analysis section (page 10, lines 232-236).

Methods

Line 191-203, Page 8: What are qualitative data? Which obey normality and which do not obey? Please provide a detailed explanation. Is there any missing data? If not, please explain how to carry out quality control, etc. If so, please explain how to handle it, such as simple imputation or multiple imputation?

Response 5: Thank you for your questions. We have divided our response into three parts: 

1) What are qualitative data?

The qualitative data is gathered from all the questions in sections A. Personal details, H. Background information, and I. Work and the Future. In addition, separate questions C9-C13, F4, G2, G3, G8, G9-G12 are qualitative. These questions are valuable for the respondents to reflect on, but they also provide useful additional information on the respondent’s situation for the service professional. We have added this description to text (page 8, lines 172-175). The development of the Abilitator is described in detail in: Wikström et al. (2020) “Development and content validity of the Abilitator: a self-report questionnaire on work ability and functioning aimed at the population in a weak labour market position” BMC Public Health. https://doi.org/10.1186/s12889-020-8391-8

2) Which obey normality and which do not obey? Please provide a detailed explanation.

As we use MLR estimation, which is robust to non-normality, the skewness for continuous data is handled when there is no floor or ceiling effects present. We have mentioned this in the Results and Discussion section (page 14, lines 311-314) 

3) Is there any missing data? If not, please explain how to carry out quality control, etc. If so, please explain how to handle it, such as simple imputation or multiple imputation?

Yes, there was missing data (page 14, lines 309-311). The percentages of missing values were 0.92% for the whole sample, 0.76% for “C. Inclusion, 1.81% for “D. Mind”, 0.73% for “E. Everyday life”, 0.52% for “F. Skills”, and 0.45% for “G. Body”. Missing data was handled with the Missing data at random (MAR) which is a default option in Mplus (page 11, lines 248-249). MAR is the same as Full information maximum likelihood (FIML) in which includes all the missing data in the analysis. Therefore, we used simple imputation.

Regarding the unclear order of the use of exploratory factor analysis and confirmatory factor analysis, please add details and explain the reasons. 

Response 6: Thank you for requesting a clarification on this issue. CFA was conducted first and then EFA. We have added a sentence to make this order apparent (page 10, lines 224-225).The main focus of this study was to conduct a CFA in in accordance with the COSMIN criteria for PROMS of which the dimensions are predetermined. When developing the Abilitator our aim was not to create a data driven PROM based on a unidimensional reflective model, where items are selected based on EFA and then tested in a separate sample with CFA. The Abilitator was developed on the basis of an expert panel-based, content driven model. Our primary hypothesis assumes that this co-developed content is relevant to the target group. EFA has been utilized to examine whether there are same underlying themes between the different domains, of which it is helpful to be aware of. In this kind of self-report questionnaire, a good enough structure is a satisfactory result. More information is provided in our earlier paper Wikström et al. (2020) “Development and content validity of the Abilitator: a self-report questionnaire on work ability and functioning aimed at the population in a weak labour market position” BMC Public Health. https://doi.org/10.1186/s12889-020-8391-8

Also, are the samples for confirmatory factor analysis and exploratory factor analysis the same population? If so, why not separate? Please explain.

Response 7: CFA and EFA are conducted in the same data. We have added a sentence to clarify this (page 10, line 226-228). In this study the same data sample could be utilized, because EFA was not used to develop a reflective, unidimensional, data driven model and then utilized to confirm it. Different data samples for EFA and CFA would be relevant if this described unidimensional model was being formed in one sample and confirmed in another. 

Methods

Page 8-10: Please add a data analysis section and explain the data analysis software used, as well as detailed data analysis methods or steps. It is best to separate research design from data analysis.

Response 8: Thank you for this suggestion. We have now divided the Methods section into Research design, Data analysis and an additional Ethics statement section, which was a request from PLOS ONE (pages 9-12, lines 212-290). We have also added descriptions on the data analysis (page 11, lines 247-251 and 262-263 and also page 12, lines 283-284)The software used i.e., Mplus is described in detail in reference 46. Muthén L, Muthén B. In Mplus user's guide (8 ed.). Los Angeles, CA, USA: Author. Available from: http://www.statmodel.com/

Results and discussion

Study population characteristics

Table 1, Page 12: If there are no special requirements for the magazine, please use a three-line form. 

Response 9: The former Table 1, now Table 2 has been modified in line with PLOS ONE’s style requirements (page 13). 

Line 266, Page 12: Is this a note for the table? Please pay attention to the formatting details, don't you even have a corner marker. 

Response 10: Yes, those were footnotes for Table 2. Unfortunately, the lines moved to next page when the pdf of the manuscript was formed. We hope this issue doesn’t occur again. We have also decreased the font size of the foot notes to make them clearer. 

Confirmatory factor analysis

Table 2, Page 13: If there are no special requirements for the magazine, please use a three-line form. 

Response 11: The former Table 2, now Table 3 has been modified in line with PLOS ONE’s style requirements (page 15). In addition, the heading of this whole section has been renamed to Confirmatory and Exploratory Factor Analysis (page 14, line 308)

Table 3, Page 14: This icon is not aesthetically pleasing, please beautify your results.

Response 12: We were considering very hard how to make Table 4 (formerly Table 3), page 15 more aesthetically pleasing without losing any of its content but were able to make only very small modifications. We are happy to take further suggestions about how to make the table look better. 

Figures: The clarity of the images is relatively low. Please pay attention to improving your resolution according to the magazine's requirements.

Response 13: This is true. These images turned out blurry when the original pdf files were transformed to the format required by PLOS ONE. If this problem persists, we will do our best to sort it in cooperation between the experts of PLOS ONE and our organization by the time of publication.

Discussion of the results

Page 17: Is it supported by the literature?

Response 14: Thank you for requesting this information. We have added a few sentences and a separate paragraph to expand on this issue (page 19-20, lines 408-432)

Significance of the results

Page 17: Please increase the discussion on the limitations of this study, including potential biases and sources of uncertainty, and provide a reflection on this study.

Response 15: We have expanded on this with a subsection titled “Strengths and limitations” (pages 21-22, lines 467-492) We also added discussion on the significance of the results (pages 20-21, lines 434-465). 

Overall

The results and discussion sections should be separated. The results are only objectively presented based on the analysis data of this time. The logic of this section needs to be strengthened.

Response 16: We have tried to strengthen the content of the Results and Discussion section as per your above suggestions as well as divided this section to subsections. However, we did not separate these two sections. We have followed the PLOS ONE’s submission guidelines in which combining is one of the given options for authors to choose: 

“Results, Discussion, Conclusions. These sections may all be separate or may be combined to create a mixed Results/Discussion section (commonly labeled “Results and Discussion”) or a mixed Discussion/Conclusions section (commonly labeled “Discussion”). These sections may be further divided into subsections, each with a concise subheading, as appropriate. These sections have no word limit, but the language should be clear and concise.” Submission Guidelines | PLOS ONE

Conclusions

Page 18: Please explain the practical significance of the research results.

Response 17: We have added an extra sentence to the Conclusions section (Page 22, lines 494-501) and several sentences to the Significance of the Results section (see Response 15) to explain the practical significance of the results. 

Overall, I feel that your writing is not rigorous enough and your logic needs to be strengthened. The biggest issue is the research design and data analysis methods section. I think it's not enough to support publication.

Response 18: We have revised the content of these sections according to your excellent suggestions (page 9-12, lines 213-284). Our goal was to explain all the methods, phases and results of this study as clearly as possible so that even a person who is not familiar with the subject and these specific statistical analysis methods could understand this relatively complex study. We hope that our revisions have made our manuscript clearer and strong enough to support its publication. 

Reviewer: 2

Ingenious way to calculate structural validity: through CFA with all concepts in the same model and considered separately.

Response 1: We highly appreciate this encouraging feedback. 

Line 269 - it seems that instead of 136 it is 132.

Response 2: Thank you very much for noticing our mistake. We have now corrected the value from 136 to 132 (3x44),(page 14, line 310). 

Table 3 - I think it would be important that factor loadings to be reproduced numerically.

Response 2: A good suggestion, thank you. We have now added the numerical factor loadings to Table 4 which was previously Table 3 (page 16). 

It does not appear out of nowhere how the questionnaire was completed by the weak labor market position population? Online? 

Response 3: Thank you for bringing this up. We have added the description under the subsection “Study sample” (page 9, lines 194-210 ).

Attachment Submitted filename: Response to Reviewers.docx

# Peer review: Decision Letter 1

**DOI:** 10.1371/journal.pone.0300182.r003

**Authors:** Leona Cilar Budler

8 Feb 2024

PONE-D-23-26849R1Examination of the structural components of the Abilitator – a self-report questionnaire on work ability and functioning aimed at the population in a weak labour market positionPLOS ONE

Dear Dr. Wikström,

Thank you for submitting your manuscript to PLOS ONE. After careful consideration, we feel that it has merit but does not fully meet PLOS ONE’s publication criteria as it currently stands. Therefore, we invite you to submit a revised version of the manuscript that addresses the points raised during the review process.

Please submit your revised manuscript by Mar 24 2024 11:59PM. If you will need more time than this to complete your revisions, please reply to this message or contact the journal office at plosone@plos.org. When you're ready to submit your revision, log on to https://www.editorialmanager.com/pone/ and select the 'Submissions Needing Revision' folder to locate your manuscript file.

Please include the following items when submitting your revised manuscript:

- A rebuttal letter that responds to each point raised by the academic editor and reviewer(s). You should upload this letter as a separate file labeled 'Response to Reviewers'.
- A marked-up copy of your manuscript that highlights changes made to the original version. You should upload this as a separate file labeled 'Revised Manuscript with Track Changes'.
- An unmarked version of your revised paper without tracked changes. You should upload this as a separate file labeled 'Manuscript'.

If you would like to make changes to your financial disclosure, please include your updated statement in your cover letter. Guidelines for resubmitting your figure files are available below the reviewer comments at the end of this letter.

If applicable, we recommend that you deposit your laboratory protocols in protocols.io to enhance the reproducibility of your results. Protocols.io assigns your protocol its own identifier (DOI) so that it can be cited independently in the future. For instructions see: https://journals.plos.org/plosone/s/submission-guidelines#loc-laboratory-protocols. Additionally, PLOS ONE offers an option for publishing peer-reviewed Lab Protocol articles, which describe protocols hosted on protocols.io. Read more information on sharing protocols at https://plos.org/protocols?utm_medium=editorial-email&utm_source=authorletters&utm_campaign=protocols.

We look forward to receiving your revised manuscript.

Kind regards,

Leona Cilar Budler

Academic Editor

PLOS ONE

Journal Requirements:

Please review your reference list to ensure that it is complete and correct. If you have cited papers that have been retracted, please include the rationale for doing so in the manuscript text, or remove these references and replace them with relevant current references. Any changes to the reference list should be mentioned in the rebuttal letter that accompanies your revised manuscript. If you need to cite a retracted article, indicate the article’s retracted status in the References list and also include a citation and full reference for the retraction notice.

**Additional Editor Comments:**

There are minor comments that need to be resolved

[Note: HTML markup is below. Please do not edit.]

Reviewers' comments:

Reviewer's Responses to Questions

**Comments to the Author**

1. If the authors have adequately addressed your comments raised in a previous round of review and you feel that this manuscript is now acceptable for publication, you may indicate that here to bypass the “Comments to the Author” section, enter your conflict of interest statement in the “Confidential to Editor” section, and submit your "Accept" recommendation.

Reviewer #1: (No Response)

Reviewer #2: All comments have been addressed

**********

2. Is the manuscript technically sound, and do the data support the conclusions?

The manuscript must describe a technically sound piece of scientific research with data that supports the conclusions. Experiments must have been conducted rigorously, with appropriate controls, replication, and sample sizes. The conclusions must be drawn appropriately based on the data presented. 

Reviewer #1: Partly

Reviewer #2: Yes

**********

3. Has the statistical analysis been performed appropriately and rigorously? 

Reviewer #1: Yes

Reviewer #2: Yes

**********

4. Have the authors made all data underlying the findings in their manuscript fully available?

The PLOS Data policy requires authors to make all data underlying the findings described in their manuscript fully available without restriction, with rare exception (please refer to the Data Availability Statement in the manuscript PDF file). The data should be provided as part of the manuscript or its supporting information, or deposited to a public repository. For example, in addition to summary statistics, the data points behind means, medians and variance measures should be available. If there are restrictions on publicly sharing data—e.g. participant privacy or use of data from a third party—those must be specified.

Reviewer #1: Yes

Reviewer #2: Yes

**********

5. Is the manuscript presented in an intelligible fashion and written in standard English?

PLOS ONE does not copyedit accepted manuscripts, so the language in submitted articles must be clear, correct, and unambiguous. Any typographical or grammatical errors should be corrected at revision, so please note any specific errors here.

Reviewer #1: Yes

Reviewer #2: Yes

**********

6. Review Comments to the Author

Please use the space provided to explain your answers to the questions above. You may also include additional comments for the author, including concerns about dual publication, research ethics, or publication ethics. (Please upload your review as an attachment if it exceeds 20,000 characters)

Reviewer #1: ID: PONE-D-23-26849R1

Title: Examination of the structural components of the Abilitator – a self-report questionnaire on work ability and functioning aimed at the population in a weak labour market position

Thank you for providing a chance to review this manuscript.

Recommendation: Minor revision.

Detailed information:

Abstract

Objective：There are too many words, please briefly summarize the research background and purpose.

Materials and Methods

The title is quite confusing, please pay attention to the subordinate relationship between the title and subtitle.

The previous review comments were well revised, but there are still a few parts that need to be improved. Please review the expression of the article as much as possible from the reader's perspective.

Thank you and my best,

Your reviewer

Reviewer #2: I think the manuscript improved after the changes made by the authors. I consider it a robust piece of research that contributes to the assessment literature by introducing this auxiliary tool necessary in the evaluation of the population in a weak labor market position.

**********

7. PLOS authors have the option to publish the peer review history of their article (what does this mean?). If published, this will include your full peer review and any attached files.

If you choose “no”, your identity will remain anonymous but your review may still be made public.

**Do you want your identity to be public for this peer review?** For information about this choice, including consent withdrawal, please see our Privacy Policy.

Reviewer #1: No

Reviewer #2: No

**********

[NOTE: If reviewer comments were submitted as an attachment file, they will be attached to this email and accessible via the submission site. Please log into your account, locate the manuscript record, and check for the action link "View Attachments". If this link does not appear, there are no attachment files.]

While revising your submission, please upload your figure files to the Preflight Analysis and Conversion Engine (PACE) digital diagnostic tool, https://pacev2.apexcovantage.com/. PACE helps ensure that figures meet PLOS requirements. To use PACE, you must first register as a user. Registration is free. Then, login and navigate to the UPLOAD tab, where you will find detailed instructions on how to use the tool. If you encounter any issues or have any questions when using PACE, please email PLOS at figures@plos.org. Please note that Supporting Information files do not need this step.

# Author comment: Author response to Decision Letter 1

**DOI:** 10.1371/journal.pone.0300182.r004

13 Feb 2024

Academic Editor’s Comments to Author:

After careful consideration, we feel that it has merit but does not fully meet PLOS ONE’s publication criteria as it currently stands. Therefore, we invite you to submit a revised version of the manuscript that addresses the points raised during the review process.

Response 1: Thank you for these encouraging comments. We have tried our best to revise the paper according to the points raised. 

Reviewers’ Comments to Author:

Reviewer: 1

Thank you for providing a chance to review this manuscript.

Response 1: Thank you for your review and helpful comments.

Abstract

Objective: There are too many words, please briefly summarize the research background and purpose.

Response 2: Thank you for this good suggestion. We have now made changes to Objectives (page 2 lines 24-29) 

Objectives 

According to the Consensus-based Standards for the selection of health Measurement Instruments (COSMIN) panel, structural validity describes how well Patient-Reported Outcome Measures’ (PROM) scores reflect the dimensions of the measured construct. The main purpose of this study was to examine the structural components of the Abilitator, a co-developed self-report questionnaire on work ability and functioning for the population in a weak labour market position.

Materials and Methods

The title is quite confusing, please pay attention to the subordinate relationship between the title and subtitle.

Response 3: Thank you for this very useful comment. We have tried to follow the PLOS ONE’s submission guidelines regarding the “Materials and Methods” section that the journal requires, see Submission Guidelines | PLOS ONE. However, we have made some changes to headings based on your suggestion. First, we unified the formatting of titles so that this article contains only either level 1 headings (Bold type, 18 pt font) for major sections or level 2 headings (Bold type, 16 pt font) for sub-sections of major sections. Second, we deleted the heading “Methods” (marked copy page 9, line 212), which partly duplicated the major heading “Materials and Methods”. We hope these changes address the points you raised. 

The previous review comments were well revised, but there are still a few parts that need to be improved. Please review the expression of the article as much as possible from the reader's perspective.

Response 4: We highly appreciate your feedback. We have tried our best to review the expression of the article from the reader’s perspective and made some amendments particularly to the Significance of the results section (pages 20-21, lines 433-461, marked copy pages 20-21, lines 437-469) and the Strengths and limitations section (page 21-22, lines 463-488, marked copy pages 21-22, lines 471-496). We also deleted two sentences in the Study sample section (marked copy page 9, lines 204-208) as they repeated the Ethics Statement (page 12, lines 285-289, marked copy pages 12-13, lines 291-295). 

Reviewer: 2

I think the manuscript improved after the changes made by the authors. I consider it a robust piece of research that contributes to the assessment literature by introducing this auxiliary tool necessary in the evaluation of the population in a weak labor market position.

Response 1: Thank you very much for your review and encouraging feedback.

Attachment Submitted filename: Response to Reviewers.docx

# Peer review: Decision Letter 2

**DOI:** 10.1371/journal.pone.0300182.r005

**Authors:** Leona Cilar Budler

23 Feb 2024

Examination of the structural components of the Abilitator – a self-report questionnaire on work ability and functioning aimed at the population in a weak labour market position

PONE-D-23-26849R2

Dear Dr. Wikström,

We’re pleased to inform you that your manuscript has been judged scientifically suitable for publication and will be formally accepted for publication once it meets all outstanding technical requirements.

Within one week, you’ll receive an e-mail detailing the required amendments. When these have been addressed, you’ll receive a formal acceptance letter and your manuscript will be scheduled for publication.

An invoice for payment will follow shortly after the formal acceptance. To ensure an efficient process, please log into Editorial Manager at http://www.editorialmanager.com/pone/, click the 'Update My Information' link at the top of the page, and double check that your user information is up-to-date. If you have any billing related questions, please contact our Author Billing department directly at authorbilling@plos.org.

If your institution or institutions have a press office, please notify them about your upcoming paper to help maximize its impact. If they’ll be preparing press materials, please inform our press team as soon as possible -- no later than 48 hours after receiving the formal acceptance. Your manuscript will remain under strict press embargo until 2 pm Eastern Time on the date of publication. For more information, please contact onepress@plos.org.

Kind regards,

Leona Cilar Budler

Academic Editor

PLOS ONE

Additional Editor Comments (optional):

Authos did take into account all comments and suggestions and improved paper quality. Paper is now ready to be accepted.

Reviewers' comments:

Reviewer's Responses to Questions

**Comments to the Author**

1. If the authors have adequately addressed your comments raised in a previous round of review and you feel that this manuscript is now acceptable for publication, you may indicate that here to bypass the “Comments to the Author” section, enter your conflict of interest statement in the “Confidential to Editor” section, and submit your "Accept" recommendation.

Reviewer #1: All comments have been addressed

Reviewer #2: All comments have been addressed

**********

2. Is the manuscript technically sound, and do the data support the conclusions?

The manuscript must describe a technically sound piece of scientific research with data that supports the conclusions. Experiments must have been conducted rigorously, with appropriate controls, replication, and sample sizes. The conclusions must be drawn appropriately based on the data presented. 

Reviewer #1: Yes

Reviewer #2: (No Response)

**********

3. Has the statistical analysis been performed appropriately and rigorously? 

Reviewer #1: Yes

Reviewer #2: Yes

**********

4. Have the authors made all data underlying the findings in their manuscript fully available?

The PLOS Data policy requires authors to make all data underlying the findings described in their manuscript fully available without restriction, with rare exception (please refer to the Data Availability Statement in the manuscript PDF file). The data should be provided as part of the manuscript or its supporting information, or deposited to a public repository. For example, in addition to summary statistics, the data points behind means, medians and variance measures should be available. If there are restrictions on publicly sharing data—e.g. participant privacy or use of data from a third party—those must be specified.

Reviewer #1: Yes

Reviewer #2: Yes

**********

5. Is the manuscript presented in an intelligible fashion and written in standard English?

PLOS ONE does not copyedit accepted manuscripts, so the language in submitted articles must be clear, correct, and unambiguous. Any typographical or grammatical errors should be corrected at revision, so please note any specific errors here.

Reviewer #1: Yes

Reviewer #2: Yes

**********

6. Review Comments to the Author

Please use the space provided to explain your answers to the questions above. You may also include additional comments for the author, including concerns about dual publication, research ethics, or publication ethics. (Please upload your review as an attachment if it exceeds 20,000 characters)

Reviewer #1: ID: PONE-D-23-26849R2

Title: Examination of the structural components of the Abilitator – a self-report questionnaire on work ability and functioning aimed at the population in a weak labour market position

Thank you for providing a chance to review this manuscript.

Recommendation: Accept.

There are no other suggestions. Hopefully, you'll do a careful detail check again to ensure the quality of the text.

Thank you and my best,

Your reviewer

Reviewer #2: (No Response)

**********

7. PLOS authors have the option to publish the peer review history of their article (what does this mean?). If published, this will include your full peer review and any attached files.

If you choose “no”, your identity will remain anonymous but your review may still be made public.

**Do you want your identity to be public for this peer review?** For information about this choice, including consent withdrawal, please see our Privacy Policy.

Reviewer #1: No

Reviewer #2: No

**********

# Editor report: Acceptance letter

**DOI:** 10.1371/journal.pone.0300182.r006

**Authors:** Leona Cilar Budler

7 Mar 2024

PONE-D-23-26849R2 

PLOS ONE

Dear Dr. Wikström, 

I'm pleased to inform you that your manuscript has been deemed suitable for publication in PLOS ONE. Congratulations! Your manuscript is now being handed over to our production team.

At this stage, our production department will prepare your paper for publication. This includes ensuring the following:

* All references, tables, and figures are properly cited

* All relevant supporting information is included in the manuscript submission,

* There are no issues that prevent the paper from being properly typeset

If revisions are needed, the production department will contact you directly to resolve them. If no revisions are needed, you will receive an email when the publication date has been set. At this time, we do not offer pre-publication proofs to authors during production of the accepted work. Please keep in mind that we are working through a large volume of accepted articles, so please give us a few weeks to review your paper and let you know the next and final steps. 

Lastly, if your institution or institutions have a press office, please let them know about your upcoming paper now to help maximize its impact. If they'll be preparing press materials, please inform our press team within the next 48 hours. Your manuscript will remain under strict press embargo until 2 pm Eastern Time on the date of publication. For more information, please contact onepress@plos.org.

If we can help with anything else, please email us at customercare@plos.org.

Thank you for submitting your work to PLOS ONE and supporting open access. 

Kind regards, 

PLOS ONE Editorial Office Staff

on behalf of

Dr. Leona Cilar Budler 

Academic Editor

PLOS ONE
